# A calcitonin receptor-expressing subregion of the medial preoptic area is involved in alloparental tolerance in common marmosets

**DOI:** 10.1038/s42003-022-04166-2

**Published:** 2022-11-21

**Authors:** Kazutaka Shinozuka, Saori Yano-Nashimoto, Chihiro Yoshihara, Kenichi Tokita, Takuma Kurachi, Ryosuke Matsui, Dai Watanabe, Ken-ichi Inoue, Masahiko Takada, Keiko Moriya-Ito, Hironobu Tokuno, Michael Numan, Atsuko Saito, Kumi O. Kuroda

**Affiliations:** 1 Laboratory for Affiliative Social Behavior, RIKEN Center for Brain Science, Saitama, Japan; 2 Laboratory of Physiology, Department of Basic Veterinary Sciences, Graduate School of Veterinary Medicine, Hokkaido University, Hokkaido, Japan; 3 School of Law, Senshu University, Kanagawa, Japan; 4 Department of Biological Sciences, Kyoto University, Kyoto, Japan; 5 Systems Neuroscience Section, Center for the Evolutionary Origins of Human Behavior, Kyoto University, Inuyama, Aichi Japan; 6 Department of Brain & Neurosciences, Tokyo Metropolitan Institute of Medical Science, Tokyo, Japan; 7 Department of Psychology, University of New Mexico, Albuquerque, NM USA; 8 Department of Psychology, Faculty of Human Sciences, Sophia University, Tokyo, Japan

**Keywords:** Social behaviour, Psychology

## Abstract

Like humans, common marmoset monkeys utilize family cooperation for infant care, but the neural mechanisms underlying primate parental behaviors remain largely unknown. We investigated infant care behaviors of captive marmosets in family settings and caregiver-infant dyadic situations. Marmoset caregivers exhibited individual variations in parenting styles, comprised of sensitivity and tolerance toward infants, consistently across infants, social contexts and multiple births. Seeking the neural basis of these parenting styles, we demonstrated that the calcitonin receptor-expressing neurons in the marmoset medial preoptic area (MPOA) were transcriptionally activated during infant care, as in laboratory mice. Further, site-specific neurotoxic lesions of this MPOA subregion, termed the cMPOA, significantly reduced alloparental tolerance and total infant carrying, while sparing general health and other social or nonsocial behaviors. These results suggest that the molecularly-defined neural site cMPOA is responsible for mammalian parenting, thus provide an invaluable model to study the neural basis of parenting styles in primates.

## Abstract (web summary)

Like humans, marmoset caregivers exhibit individual parenting styles. Among these, infant tolerance is in part regulated by a subregion of the medial preoptic area enriched with calcitonin receptor-expressing neurons.

## Introduction

Parenting, or primary caregiving, is of profound importance in all mammals including humans, as infants are born immature and require extensive care for development. Parental care in mammals includes the provision of maternal milk, cleaning the body, helping with locomotion, and protection from environmental hazards^1–7^. As there are no mammalian species without parental behaviors, the basic neural mechanisms required for human parental behaviors should be conserved during mammalian evolution.

The medial preoptic area (MPOA) is the most crucial brain area for parental and alloparental caregiving behaviors in a wide variety of mammals, including laboratory rats^8–10^, hamsters^11^, biparental California mice (*Peromyscus californicus*)^12^, laboratory mice^13^, rabbits^14^, and sheep^15^, and with supportive observations in humans^16^ (See in ref. ^5^ for a comprehensive review of this literature). MPOA neurons expressing estrogen receptor α or galanin mediate mainly pup retrieval and pup grooming, respectively^17–21^. To further specify the essential neurons for pup retrieval anatomically and molecularly, we have narrowed down the essential regions for infant care by mothers, fathers, and alloparents (collectively called caregivers hereafter) in laboratory mice to the central part of the posterior MPOA (cMPOA, overlaps with the posterolateral subdivision of the medial preoptic nucleus^22^)^13,23^, and then screened the candidate molecular markers and identified the calcitonin receptor (Calcr) in laboratory mice^24^. We have reported that Calcr not only serves as a marker for neurons critical for parental care in the cMPOA, but also functions molecularly to heighten maternal motivation in postpartum females. However, in non-human primates, the neural basis of infant caregiving behaviors has not been specifically investigated.

While the biological mother is the only caregiver for an infant in the vast majority of primate species, New World monkey common marmosets (*Callithrix jacchus*) exhibit a cooperative breeding system, in which non-breeding helpers assist with infant care^25–36^. Marmoset infants are generally born as twins and are almost continuously carried during the early preweaning period. Infant carrying is a high-cost behavior for this arboreal species and limits the carrier’s ability to leap^37^; thus infant carrying is shared by the mother, father, and older siblings. This unique social feature together with vocal communication^38^ makes marmosets an excellent model for studying human parenting, in which allomothering is frequent and even the norm in many societies^39,40^. Utilizing this alloparenting characteristic in marmosets, here we investigated the responsible brain area for marmoset caregiving behaviors. We identified that the Calcr-expressing MPOA neurons were significantly activated during infant care. Functional suppression of this marmoset counterpart of the mouse cMPOA led to a sharp reduction of alloparental tolerance, defined as the ability to endure infant carrying, without affecting other social and non-social behaviors that we tested. Thus, this study demonstrates the evolutionarily-conserved essential role of the Calcr-expressing subregion of the MPOA in infant care in primates.

## Results

### Outline of the experimental design and the marmoset families used in this study

**Fig. 1:**
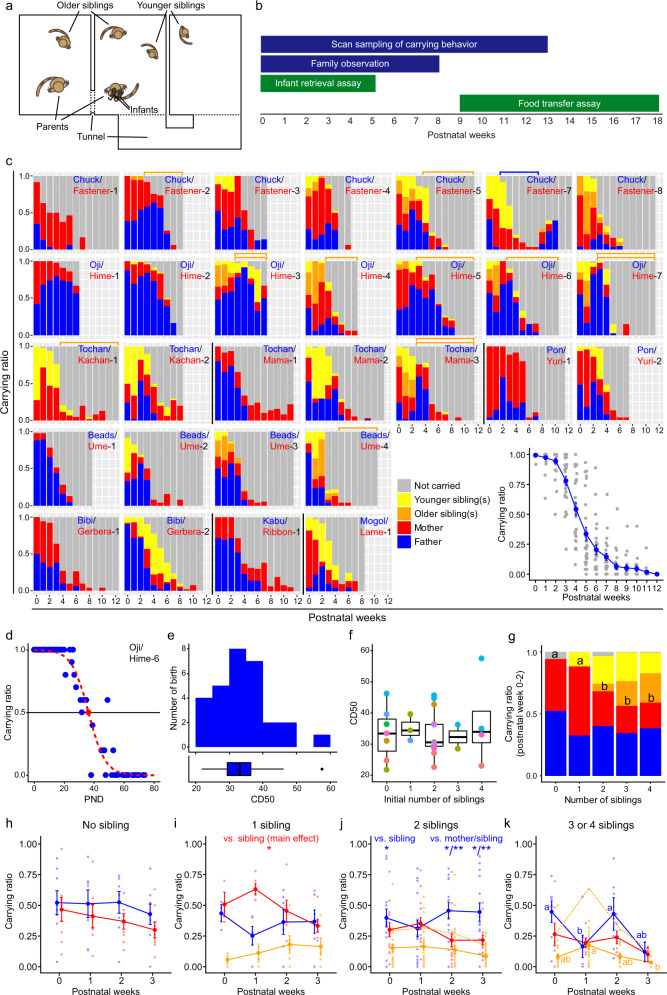
Infant carrying behavior in marmoset family. **a** A schematic illustration of a home cage-complex. Three cubicle cages were jointed with tunnels. Parents, two older siblings, two younger siblings, and two infants were kept in this cage complex. **b** Experimental timeline. After a mother’s delivery in a family, daily scan sampling of carrying behavior, the family observation, and the infant retrieval assay were started. Then the food transfer test was conducted starting from the weaning period. **c** Changes in infant carrying ratio as measured by scan sampling through postnatal weeks in 29 births from 9 families. Among the 7366 scan samples, 4699 were data with twins. Different colors indicate different caregivers. Yellow: younger sibling, Orange: older sibling, Red: mother, Blue: father, and Gray: not carried. In several births, one or two family members were separated from the rest of the family during the observation period; orange brackets indicate the weeks the older siblings were permanently removed from the family for the experimental intervention (Supplementary Data 2 in detail); a blue bracket in Chuck’s 7th birth indicates the temporary separation of Chuck and Cubby after the incidence of infant (Nibs) injury, to prevent the further attack of the infant. The line graph shows the mean±SEM carrying ratio for 29 births through the postnatal weeks. **d** An example of the daily carrying ratio (blue dots) over postnatal days in the 6th birth in the Oji’s family obtained from the scan sampling. A day on which the carrying ratio crosses 50% (CD50, red dot) was calculated using logistic-curve fitting (red dashed line). **e** Distribution of CD50 (*n* = 29 births in 9 families). The median CD50 was 33.02. This data included the 11 births in which family member(s) were removed/separated from the family (as described in Fig. 1c, Supplementary Data 2 for details). Excluding these births changed CD50 only slightly (median PND: 31.12). **f** CD50 was box-plotted over the initial number of siblings in a family at birth. Horizontal lines indicated median, top and bottom ends of boxes indicated 25th and 75th percentiles, and top and bottom ends of vertical bars indicated maximum and minimum data points within 1.5× inter-quartile range. The number of siblings did not affect CD50 (LMM, *n* = 29 birth of 9 families). Different colors indicate different families. **g** The carrying ratio of parents (father+mother) obtained from scan sampling significantly dropped with an increasing number of siblings. Different letters indicate statistically-significant differences between postnatal weeks; that is, if two variables contain the same alphabet (eg. ab and bc), the difference between these two variables is not statistically significant (binomial GLMM, *n* = 29 birth of 9 families, *p* < 0.05). The present data did not demonstrate a significant reduction in the carrying ratio either by fathers or mothers alone. Blue: fathers, red: mothers, yellow: younger siblings, and orange: older siblings. **h**–**k** Mean±SEM carrying ratios, separately plotted by the numbers of siblings in the family (**h**; no sibling, **i**; one sibling, j; two siblings, **k**; three or four siblings). Orange solid lines, average carrying ratios of individual siblings; dashed line, averaged total carrying ratios of all the siblings at each birth. The total carrying ratio by all siblings was significantly higher than the carrying ratios by the father or the mother in postnatal week 1 when there were 3 to 4 siblings in the family (LMM, *p* < 0.01). Father’s carrying ratios were significantly lower in this week than in the week 0 or 2 (LMM, different letters indicate significant difference between postnatal weeks within the same caregiver type, *p* < 0.05). Blue: fathers, red: mothers, and orange: siblings (younger and older were pooled).

Thirty-seven births from 10 marmoset families in the captive animal facility at RIKEN, Japan, consisting of 81 individuals in total, were analyzed (Supplementary Data 1, 2). In each family group, the maximal number of animals was eight, including the parents, older and younger siblings (two each at most), and twin infants. One mother (Fastener) had dysfunction of one nipple and could sustain only one infant at each birth. The families were kept in a large family cage, consisting of three cubicle cages connected with each other (Fig. 1a), except for the initial few births where they were housed in two connected cages.

Four experimental paradigms were used to analyze the interaction between the infants and other family members, starting from the day of birth (Fig. 1b)^41–43^: (1) instantaneous scan sampling of the family cage, 5 times per day from PND 0 to PND 92 (minimum and maximum data points shown in the dataset), (2) continuous 20-min focal observation of the family from PND 0 to 60, (3) dyadic infant-retrieval assay, starting from PND 1 to 41 or until the infant escaped from the mesh basket by itself, and (4) dyadic food transfer assay from PND 69 to PND 128. It should be noted that all the caregiver-infant interactions were tested within the same family members, because marmoset infants actively reject non-family caregivers unlike rodent pups.

### Family sharing of infant carrying: the role of siblings’ help

Scan sampling of carrying behavior in the family cage elucidated the total and family sharing of infant carrying in each family (Fig. 1c). The average carrying ratio of the litter, namely the height of each bar in Fig. 1c, was maintained high throughout the first three weeks and infants were almost always carried, even though there were high individual variabilities of carrying care among caregivers (i.e., the color components of each bar, see below). The carrying rate sharply declined through the fourth and fifth weeks. The median day on which the carrying ratio fell below 50% (50% carrying date, CD50) was PND 33.02 (Fig. 1d, e). This CD50 and the total carrying rate during the first three weeks for each infant were not affected by the number of siblings in a family (Fig. 1f, g), suggesting that total carrying is determined by infant needs, rather than by the number of available caregivers, and that biological parents by themselves can fulfill the infant’s needs at least in our captive environment.

Nevertheless, when the number of siblings was larger than two, the total carrying by the parents decreases significantly (Fig. 1g), consistent with the previous reports^26,44–46^. While the average carrying share by each sibling did not exceed the share of either parent (Fig. 1h–k), the sum of three-or-four sibs’ share became significantly larger than the average share of either parent at postnatal week 1 (Fig. 1k). Sex differences were not significant in siblings as shown in Supplementary Fig. 1, thus the data were combined for sibling sex. Also, when the siblings were three or four, the father’s carrying share dropped significantly in postnatal week 1, at which time the mother’s postpartum ovulation occurred (at 10.5 ± 0.7 days after delivery^47^), as reported previously^32^. The transient decrease of infant carrying by fathers was also observed in the infant retrieval assay (see below), implying the paternal motivation for infant caregiving may compete with sexual motivation. We will study this issue more in a separate study. Our data also suggest that the existence of three siblings or more may also accelerate parental reproduction, along with the reported facilitatory effect on infant survival in the wild^48^.

### Infant carrying limits the carrier’s other activities

**Fig. 2:**
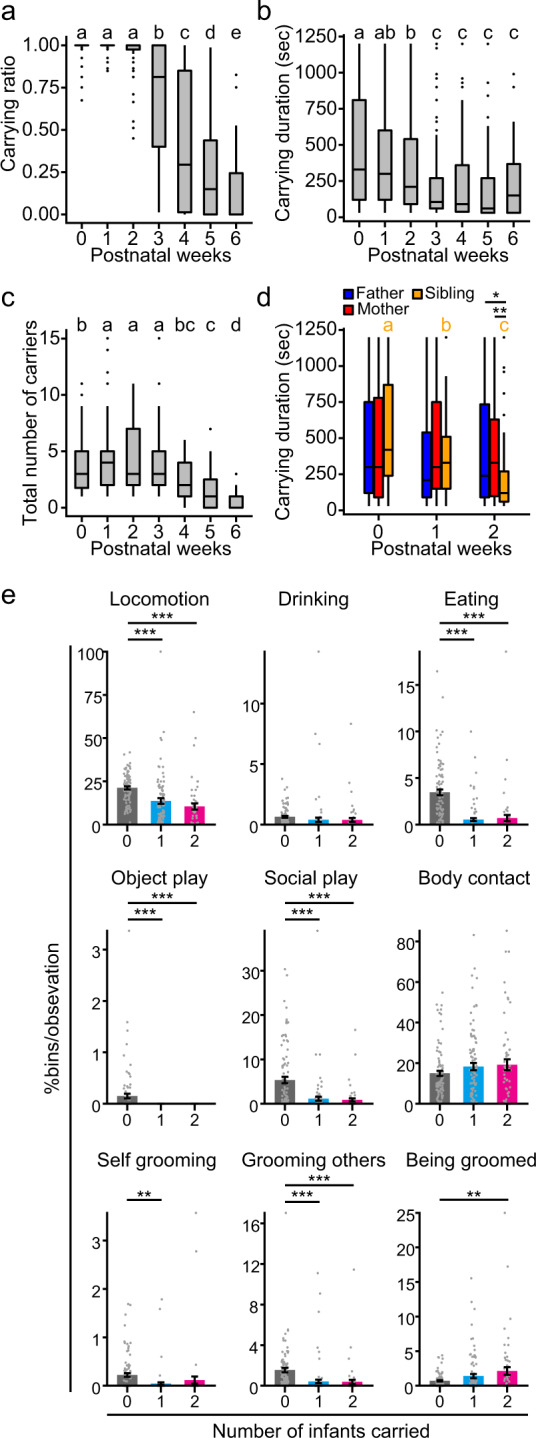
Costs of infant carrying behavior. A total of 526 20 min family observations were made for 30 births in 9 families including 48 animals. The same dataset was used for all panels within Fig. 2. For box plots (**a**–**d**), horizontal lines indicated median, top and bottom ends of boxes indicated 25th and 75th percentiles, and top and bottom ends of vertical bars indicated maximum and minimum data points within 1.5× inter-quartile range. Different letters indicate statistical significance between postnatal weeks (2a, 2b, 2d, LMM; 2c, Poisson GLMM, *p* < 0.05). Asterisks denotes statistical significance (LMM, ***p < 0.001, **p < 0.01, *p < 0.05). **a** Carrying ratios over postnatal weeks was maintained at a high level for the first 3 weeks. **b** Carrying durations over postnatal weeks. Carrying bouts with a cut-off at the beginning or end of the observation that was shorter than the median duration in each week were excluded from the analysis (see Supplementary Fig. 3, middle). Animals that received the surgical intervention were excluded from this analysis (16 animals in 14 births). Carrying duration gradually decreased within the first 3 weeks. **c** Total numbers of carriers significantly increased in PNW 1 to 3 compared to PNW 0. **d** Carrying durations of fathers, mothers, and siblings in the first three postnatal weeks. Animals that received the surgical intervention were excluded from this analysis. Only siblings significantly decreased carrying durations during the postnatal 3 weeks. Siblings’ carrying durations were significantly shorter than those of fathers and mothers in postnatal week 2. Blue: fathers, red: mothers, and orange: siblings (younger and older were pooled). **e** Mean±SEM occurrence rates of various behaviors over the number of infants carried. Occurrence rates were calculated as the number of bins that the behavior occurred in during each carrying state (not-carrying, carrying one, or two infants) divided by the number of bins of each carrying state. Data included PND 0 (minimum) to PND 60 (maximum). Animals that received the surgical intervention were excluded from this analysis (16 animals in 14 births). A significant decrease was observed in locomotion, eating, object play, social play, self-grooming, and grooming others. In contrast, being groomed significantly increased as the number of carried infants increased.

The mean body weight of infants, 29.00 ± 2.83 g (mean ± SD) at birth, increases steadily and reaches 68.68 ± 8.31 g (mean ± SD) at PND40 (Supplementary Fig. 2). At postnatal week 4, the total body weight of twins becomes approximately 30% of body weight of parents and exceeds 40% of weight of younger siblings in average. Thus, it is presumable that infant carrying is a physical burden. Indeed, data from the focal 20 min family observation (see Table 1 for a list of recorded behaviors) demonstrated that while the total carrying time for each infant is almost constant during the first three weeks (Fig. 2a), the duration of each carrying bout decreases significantly (Fig. 2b), and the number of total infant carriers during the 20 min observation period increased (Fig. 2c). The duration of each carrying bout declined most rapidly in siblings but not in fathers and mothers during the first three postnatal weeks (Fig. 2d), suggesting a larger impact of the infant’s body weight for the siblings. These data suggest that when infants become older and heavier, the family copes with the carrier’s burden by increasing the infant transfer to the other caregivers, thereby shortening the duration of each individual’s carrying bout. The carried infants are often transferred directly from one caregiver to an adjacent caregiver, or indirectly via being rejected and getting off from one caregiver, then calling vigorously to attract another caregiver for the next carry.

**Table 1:** List of observed behavior.

Sampling	Item	Description
Scan sampling	Carrying	Infant(s) cling on body. Identity of infant(s) was recorded
	Body contact	Physical contact of body parts with other marmoset other than infant(s)
	Staying cage	Cage where animal is staying (right or left)
1–0 sampling	Successful retrieval	Retrieve infant(s) from other carrier or cage floor and start carry
	Failed retrieval	Same motion as successful retrieval but infant does not cling
	Anogenital licking	Lick infant’s anogenital region to facilitate infant’s excretion
	Rejection	Carrier scratches, bites infant(s), or rubs infant(s) on cage wall/floor
	Social play	Chase, Chased by, or wrestle with other marmoset
	Object play	Manipulate objects by hand or bite objects
	Grooming others	Manipulate other marmoset’s fur by hand or teeth
	Being groomed	Groomed from other marmoset
	Self grooming	Groom own body parts
	Self scratch	Scratch own body parts by hand
	Scent marking	Rub anogenital area on cage
	Eating	Masticate food pellets
	Drink	Lick a water nozzle
	Vocalizing	Emit vocalization including twitter, phee, trill, tsik, ek, chatter, or vhee
	Stereotypy	Repeated vertical circling more than three times
	Yawn	Yawing

We then examined the cost of infant carrying by its suppressing effects on other behaviors, as a function of the number of infants being carried. It turned out that infant carrying, even of only one infant, significantly limited the caregiver’s locomotor, feeding, object play, social play, and grooming activities (Fig. 2e). Among 2380 observed instances by the scan sampling at which there were twins in the family and both twins were carried, twins were carried by the same individual in the majority of 1977 cases (83.1%), suggesting a strategy that allows other family members to move around actively for effective foraging.

Interestingly, the carrying members received more grooming care from non-carrying members (Fig. 2e), supporting the notion that extra allogrooming was given as a social reward^49^, although another study reported the lack of correlation between the received grooming and caregiving contribution in non-breeding members^50^.

### Infant retrieval assay reveals dyadic relationship between an infant and a caregiver

In addition to the above-described family observations without experimental perturbations, the infant retrieval assay^43,51^ was performed to investigate a dyadic relationship between an infant and one of the family members. The advantage of testing each caregiver and only one infant at a time enabled a clearer assessment of characteristics of each caregiver separately from family dynamics, as well as the attachment behaviors of each infant toward each caregiver (see Supplementary Movie 1).

**Fig. 3:**
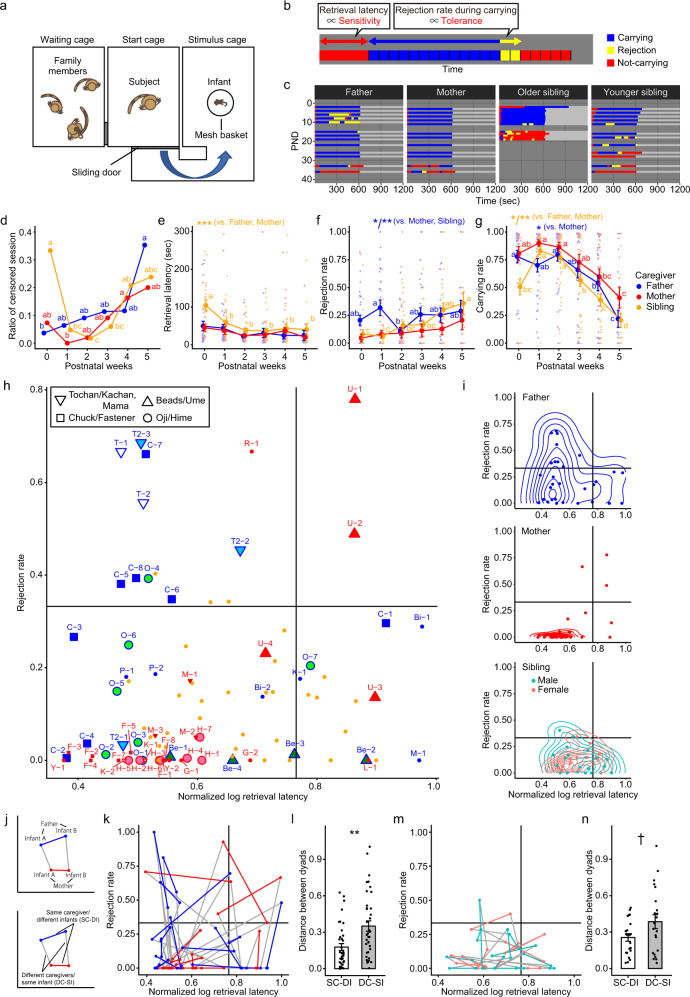
Dyadic infant retrieval assay revealed intrinsic caregiving parameters. The infant retrieval assay was conducted for 815 trials using 46 intact caregivers in 30 births of 9 families in total (Father; 277 trials, *n* = 8, Mother; 249 trials, *n* = 9, Siblings; 289 trials, *n* = 32 consisted of 19 males and 13 females, 3 of 32 were also subjected as two fathers and a mother later). The following panels were used for this dataset. Different letters indicate statistical significance between postnatal weeks within the same caregiver type (LMM, *p* < 0.05). Asterisks denotes statistical significance (LMM, ***p < 0.001, **p < 0.01, *p < 0.05, ^†^*p* < 0.1). **a** A schematic illustration of the infant retrieval assay. A caregiver can reach an infant in a metal basket through a tunnel after opening a sliding door. A retrieval latency was recorded, then the caregiver’s behavior including infant carrying behavior was observed for 10 min. **b** An example raster plot of the infant retrieval assay. blue, the caregiver carried the infant at the beginning of the bin; yellow, the bins of which beginning the caregiver carried the infant but rejected it within the bin; and red, the caregiver did not carry the infant at the beginning of the bin, or before the initial retrieval. The length leftmost red bar indicates the retrieval latency, which reflects the caregiver’s parental sensitivity to signals emitted by the infant (e.g., distress vocalization). The rejection rate is calculated as the number of bins with rejection (yellow) divided by the sum of the number of bins with carrying (blue) and rejection (yellow). It can be considered as an inverse proportion of parental tolerance. **c** A typical example of the whole-family results of the infant retrieval assay shown as a raster plot through postnatal days. Each bar corresponds to one assay as described in (**b**). Note that the older sibling here received NMDA injections into the MPOA on PND 11 for the lesion experiment described later. He and also other lesioned subjects were not included in the analyses in this figure. **d** A ratio of sessions during which retrieval behavior did not occur for 300 s. Sessions with a longer retrieval latency than 300 s. were treated as censored sessions at 300 s. within this figure to match to initial cut-off latency (see methods). Blue: fathers, Red: mothers, Orange: siblings. Asterisks indicate statistical significance between caregiver types (fathers, mothers, siblings). Different letters indicate statistical significance between postnatal weeks within the same caregiver type (Fisher’s exact test, *p* < 0.05). Note that statistical power in PNW 5 was lower than in other PNWs due to a lower number of trials (17, 15, and 21 trials for father, mother, and siblings, respectively). **e** Mean±SEM retrieval latencies which did not include censored sessions. Blue: fathers, Red: mothers, Orange: siblings. **f** Mean±SEM rejection rates after successful retrieval. The rejection rate was calculated as the number of bins with rejection / (carrying+rejection). Blue: fathers, Red: mothers, Orange: siblings. **g** Mean±SEM carrying rates, calculated as (a number of bins with (carrying+rejection) ×30) / total session time (sec). Note that retrieval latency is included within the denominator. **h** Plots for each caregiver’s retrieval latency and rejection rate at each birth (i.e., one dot represents each parent at one birth, by averaging these parameters for the litter infants when two infants from the same birth were alternately used for the assay) during the first four weeks. The data from the first week for the caregivers that had no experience with the infant retrieval assay previously were excluded. Representative fathers (Tochan, Chuck, Oji, Beads) and mothers (Hime and Ume) were shown by large markers. Father and mother’s names were shown for each data point as an initial letter. Numbers after their names indicate the number of births. Other data points shown in blue, red, and orange indicated fathers, mothers, and siblings, respectively. Vertical and horizontal lines indicated mean + 1 SD for each axis. The Pearson product-moment correlation coefficient *r* = 0.08, *p* = 0.44, n.s. **i** Datapoints of Fig. 3h are shown separately for fathers (blue), mothers (red), and siblings (blue-green: males, pink: females). Two-dimensional probability density was overlayed as a contour with 10 bins. **j** The schema shows the method to compare the two parenting parameters of the same caregiver with two different infants (SC-DI, colored segments) and of the same infant with its two different caregivers (DC-SI, gray segments) on the two-dimensional plots of the average retrieval latency and rejection rates for each dyad (Fig. 3k and m). **k** Two-dimensional plots of two parameters of each parent-infant dyad. Births with only one infant were excluded from this plot. 73.7% (28 out of 38) of colored segments, and 44.7% (17 out of 38) of gray segments were in one quadrant. **l** Mean±SEM lengths of SC-DI and DC-SI of Fig. 3k. The SC-DI differences are significantly shorter than the DC-SI differences (Welch’s two-sample *t*-test, *n* = 38 each, **p < 0.01). **m** Two-dimensional plots of two alloparenting parameters of each younger sibling-infant dyad (blue-green: male siblings, pink: female siblings). Births with two siblings were selected for this plot. **n** Mean±SEM lengths between SC-DI and DC-SI of Fig. 3m. The SC-DI differences are marginally shorter than the DC-SI differences (Welch’s two-sample *t*-test, *n* = 22 each, ^†^*p* < 0.1).

The marmoset family was housed in a cage complex (Fig. 1a) which consisted of three cages (rooms) connected with each other. On the test day, the family members other than the subject dyad were contained in the left room, the subject caregiver was in the center room, and the infant was placed in a small basket with a cloth-covered heater in the right room. The center and right rooms were connected by a removable mesh tunnel, initially shuttered at the entrance of the center room. The infant retrieval assay started by opening the shutter (Supplementary Movie 1 and Fig. 3a); typically, the isolated infant constantly emitted vocalizations and the caregiver entered the infant cage and approached the infant in the basket. When the caregiver leaned their upper body into the basket, the infant clung to the caregiver, and this was coded as a successful retrieval. The time from the start of this assay to the successful retrieval was recorded as the retrieval latency. From this time-point, the caregivers’ behaviors were coded on-site for 10 min with 30 s bins (Fig. 3b), and mutual interactions were coded off-site in more detail using video and audio recordings. The caregiver carried the infant for a while and during this period infant vocalizations were reduced (Supplementary Movie 1). Eventually, the caregiver may start to reject the infant, by biting, rubbing-off or rolling over the carried infant to remove the infant from the body. The infants typically respond to these rejections by vigorous vocalizations and by getting off from the caregiver (Supplementary Movie 2). After rejection and removal of the infant, the caregiver may retrieve the infant again by approaching and allowing the infant to cling to its body.

Each assay result is visualized as a colored bar in 30 s bins (Fig. 1b): blue, the bins of which beginning the caregiver carried the infant, and during which the caregiver did not reject the infant; yellow, the bins of which beginning the caregiver carried the infant and rejected it within the bin; and red, the un-binned retrieval latency period from the assay start to the initial retrieval, and the bins of which beginning the caregiver did not carry the infant.

The on-site observation coding yielded three caregiving parameters, (i) the retrieval latency (s), as depicted as an initial red part in Fig. 3b; (ii) rejection rate, the number of bins that included rejection (yellow) divided by the number of bins of infant carrying (yellow + blue); (iii) carrying rate, the total duration of infant carrying (yellow + blue) divided by the total length of the session (retrieval latency+600 s). All the caregivers in the family were tested at least 1 time per week, and repeatedly along with the infant’s development, from PND 1 to 41 (Fig. 3c). And for each caregiver, it’s retrieval assay sessions with each twin at one birth-time were shown in one matrix (Fig. 3c, summarizing the whole-family results obtained in the 5th birth of Oji’s family), because neither the infant’s sex nor its individuality affected caregivers’ behavior toward the infant significantly more than the caregivers’ intrinsic characteristics (Supplementary Fig. 4, see also below Fig. 3j–n). Since the siblings’ sex did not significantly affect caregiving parameters (Supplementary Fig. 1), the sibling data were combined for sex in each of the following analyses.

### Caregiving parameters change along with infant development and are different among mothers, fathers and siblings

For mothers and fathers, the ratios of censored sessions (Fig. 3d), that is, the sessions in which the caregiver never retrieved the infant, were low in the early postnatal period and gradually increased through subsequent postnatal weeks. After excluding these censored sessions, the retrieval latency (Fig. 3e) was relatively constant throughout the developmental period, suggesting the binary manner of caregiver’s choice to retrieve or not to retrieve the infant. The infant rejection rate (Fig. 3f) did not increase along with infant age in mothers and fathers. The total carrying rate (Fig. 3g) remained high during the first three weeks, and gradually decreased from postnatal week 3, consistent with the carrying ratios observed in the home cage (Figs. 1c, 2a).

Siblings showed a higher ratio of censored sessions and longer retrieval latencies, and a low carrying rate in the first week (Fig. 3d, e, g). This was due to inexperience with the test and a lack of skills to retrieve the infant from the basket, because their latency rapidly improved after the second week, and became indistinguishable from the parents afterwards (Fig. 3d, e, Supplementary Fig. 5). Thus, caregiving experiences shortened retrieval latency in caregiving-naïve individuals. Siblings also increased their infant rejection rate (Fig. 3f) significantly along with infant development, and exhibited a more rapid decline in the total carrying rate than parents (Fig. 3g), consistent with the larger impact of infant growth on siblings’ carrying duration (Fig. 2d). In addition, the significantly higher rejection rate in fathers compared to mothers and siblings in postnatal week 1 was observed together with the low carrying ratio in the family cage (Fig. 1k), supporting the notion that the paternal caregiving and male sexual motivations are competitive. In contrast, mothers did not decrease carrying during the same period either in social or dyadic situations.

The average retrieval latency and rejection rate of each caregiver at each birth during the first four weeks were not correlated with each other, nor with the combination of caregiver-infant sex (Fig. 3h, Supplementary Fig. 4). Mapping these two parameters separately for fathers, mothers, and siblings (Fig. 3i) illustrated the general tendency of caregiving features in these groups; the majority of mothers show short retrieval latency and low rejection, while fathers are more variable, and the siblings were, unexpectedly, intermediate regardless of sex. These data indicate more intense caregiving in mothers than in fathers in the dyadic situation, which is discrepant from the data showing an equal contribution by parents in the family situation (Fig. 1h–k); for example, in the family of Oji (father) and Hime (mother), Oji carried infants more than Hime in the family situation (Fig. 1c, the second row), except at the fourth birth. On the other hand, Hime showed more caregiving in the dyadic infant retrieval assays than Oji (Fig. 3h, compare red (Hime) and green (Oji) circles). This discrepancy may be caused by the family dynamics or social decision-making of infant carrying in the marmoset family; for example, mothers may reduce their contribution when other caregivers are available, but would carry longer if the infant is not carried and distressed otherwise (see in ref. ^52^ for *Callithrix kuhlii*). Another possibility is that fathers would increase their caregiving share when the mating partner is around, either proactively aiming for a mating opportunity or because they are solicited by the other members, in particular, the mother (however, see in ref. ^53^).

### Individual variability in parenting and alloparenting styles: sensitivity, tolerance, and quantity

In each caregiver, the rejection rate and the retrieval latency in the infant retrieval assays are relatively constant across birth and litters (Fig. 3h), consistent with the previous literature^25^. For example, while the mother Hime (red circles in Fig. 3h. Each symbol corresponds to Hime’s caregiving parameters at one birth) always showed low rejection and retrieval latencies, Ume (red triangles in Fig. 3h) showed high values for both parameters. The father Beads (green triangles) showed modestly high retrieval latencies and very low rejection rates, while Tochan (blue inverted triangles) showed low retrieval latencies and high rejection rates, except for the first birth of his second mating partner, Mama (a blue inverted triangle at the bottom of the panel, labeled T2-1). These consistent patterns were observed in the family scan sampling.

As infant carrying involves both a caregiver and infants, retrieval latency and rejection rate may also be dependent on infants’ characteristics, such as the amount of infant separation calls or the infant’s manner of clinging to the caregiver’s body. To compare the contribution of each parent and each infant to these caregiving parameters, a new plot for dyadic relations (i.e., one dot represents the parameters of each parent with each infant averaged during the first 4 weeks, Fig. 3j, k) was created. Then, the difference of these parameters between two infants for the same parent (open bar, Fig. 3l) and between two parents for the same infant (filled bar, Fig. 3l) was compared. A significantly larger difference was detected for these parameters in the two parents with the same infant than in the two infants with the same parent, suggesting that parents contributed more to the caregiving parameters than infants. For the family containing more than two siblings, we also compared the contribution of each sibling and each infant on the retrieval latency and the rejection rate (Fig. 3m, n), and found marginally (*p* = 0.0545) larger variations between different alloparents for the same infants than between different infants for same alloparents (Fig. 3m, n). These data suggest that the caregiving parameters in a given dyad are determined more by the caregiver than by the infant.

Moreover, across different social settings, there were significant correlations between the relevant parameters of infant-directed behaviors in each caregiver (see below). Namely, the carrying ratio in family scan sampling had a strong correlation with the total carrying duration in family 20 min observation, and a mild correlation with the carrying rate in the retrieval assay. The rejection rate in the family 20 min observation had a positive correlation with the rejection rate in the infant retrieval assay. These data confirmed the persistent and intrinsic caregiving characteristics in each animal.

With these observations, we define the following indices of parenting and alloparenting styles in the infant retrieval assay; the sensitivity of the caregiver towards infant distress vocalizations during separation (Supplementary Movie 1) is defined as (the total session time – retrieval latency) / total session time; and the tolerance of the caregiver to infant carrying as (1 – rejection rate). The quantity of infant caring behavior is defined as the carrying rate (total carrying amount during the session), and is the function of both sensitivity and tolerance.

### Food transfer ratio to juvenile offspring as another form of infant care

**Fig. 4:**
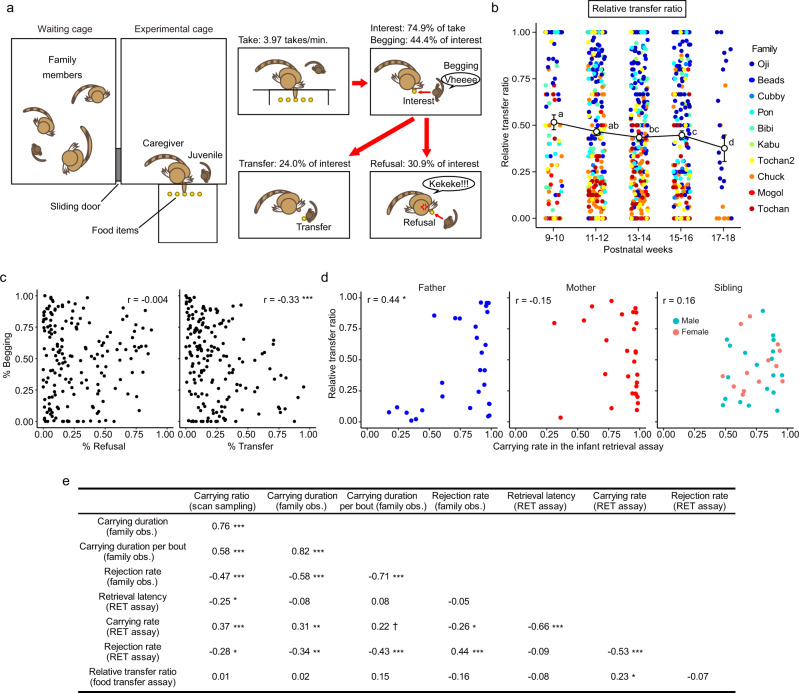
Food transfer test and correlations of indices among observations/assays. The food transfer assay was conducted for 969 sessions using 53 intact caregivers in 35 births of 10 families in total (Father; 318 trials, *n* = 9, Mother; 317 trials, *n* = 10, Siblings; 334 trials, *n* = 38 (24 males and 14 females), 4 of 38 were also subjected as three fathers and a mother later). Twenty-one out of 969 sessions (2.2%) were excluded from the analysis because neither transfer nor refusal occurred during the session. The following panels used this dataset. **a** The setting of the food transfer test. **b** Mean±SEM relative transfer ratio for each caregiver for each birth, calculated as the number of food transfers divided by the number of food transfers plus refusal, significantly decreased through postnatal weeks (LMM, different letters indicated statistical significance between postnatal weeks, *p* < 0.05). Datapoints are color-coded for each family. **c** Correlations between infant’s begging rate and refusal/transfer rates among each dyad. A significant negative correlation was revealed between infant’s begging and transfer rate. (Pearson’s correlation coefficient, *r* = −0.33, ***p < 0.001). **d** Correlations between relative transfer ratio and carrying rate in the infant retrieval assay. Fathers showed a significant positive correlation (Pearson’s correlation coefficient, *r* = 0.44, *p < 0.05). **e** Pearson’s correlation coefficients among major indices of caregiving behaviors measured in the present study (94 data points using 46 intact caregivers in 30 births of 9 families. Father; 30 datapoints, *n* = 8, Mother; 29 datapoints, *n* = 9, Siblings: 35 datapoints, *n* = 22). To exclude the influence of infant development, the parameters were calculated from the data during the first three weeks, except for the food transfer. Note that for the retrieval assay, trials with retrieval latency longer than 300 s. were treated as censored sessions for this analysis to match the initial cut-off time. (***p < 0.001, **p < 0.01, *p < 0.05, ^†^*p* < 0.1. *p* values in this table were adjusted with the Benjamini-Hochberg method due to a large number of paired comparisons).

Marmoset infants wean at around postnatal 6 weeks in captivity, and 8–10 weeks in the wild^54^. Food transfer to juveniles provides them with additional nutrients during weaning, and such behavior is observed in a limited number of primate species, which includes common marmosets^29,42,55^. We performed food transfer assays in a dyadic setting for a caregiver and its juvenile offspring at PND 69 to PND 128, utilizing one compartment of their home cage-complex (Fig. 4a). Familiar food items (steamed sweet potato cubes or rice cereals) are presented at a distance reachable for the caregiver but not for the juvenile. Once a food piece is taken by the caregiver, the juvenile often approaches the caregiver, shows interest in the food, produces begging calls, and tries to fetch the food from the caregiver’s hand. The food is successfully transferred when the caregiver allows the juvenile to take the food (Supplementary Movie 3). The caregiver may instead refuse transfer, by running off and/or vocalizing aggressively toward the juvenile (Supplementary Movie 4). In other instances, the caregiver finishes or drops the food piece before transfer or refusal, or the juvenile loses interest and moves away.

The relative food transfer ratio for caregivers, calculated as the number of successful transfers / (transfers+refusals) per session, was higher when the offspring were younger and decreased along infant development (Fig. 4b), implying that the caregivers were generally more tolerant toward younger juveniles, this finding being consistent with a previous report^42^. Caregivers in the Oji and Beads families showed high, and caregivers in the Tochan and Chuck families showed low relative transfer ratios.

The infants’ begging calls negatively correlated with the transfer (Fig. 4c), suggesting that the caregivers were not solicited to be tolerant by begging, and that begging was sustained simply when the caregiver did not allow food transfer. The relative transfer ratio did not differ among fathers, mothers, and siblings, but appeared to be inherent to the individual, and showed a mild positive correlation with the carrying rate during infant retrieval assay, particularly in fathers (Fig. 4d, e).

### Consistency of parenting and alloparenting styles across settings and testing

Bivariate correlation analysis among the above-described major parameters of each caregiver at each birth obtained from three different experimental paradigms listed in Fig. 1b revealed reasonable correlations between the similar caregiving parameters across settings (Fig. 4e); for example, within quantity of infant care behaviors parameters, carrying ratio obtained from scan sampling strongly correlated with total carrying duration in 20 min family observations, and modestly with carrying rate in dyadic infant retrieval assays, the latter possibly due to different social dynamics. The rejection rate in family observations correlated significantly with the rejection rate in infant retrieval assays, and most prominently, negatively with carrying duration per bout in family observation, suggesting the contribution of infant rejection to ending the carrying bout. The retrieval latency in infant retrieval assays showed limited correlations except for carrying rate in the same setting, suggesting that this parameter is rather independent of other parameters.

### The Calcr-expressing neurons in the marmoset cMPOA are activated during infant care

To elucidate the brain area critical for infant caregiving in common marmosets, non-breeding older siblings, but not breeding parents were subjected to invasive neurological analyses, based on two reasons; first, as shown above, older siblings exhibit comparable infant carrying as that observed in parents; and second, the responsible brain areas for maternal, paternal and alloparental behaviors are shown to be essentially the same in the rodent studies^5,13,23,24,56^ (but in ref. ^57^). Therefore, in this study, only the older siblings were subjected to invasive analyses, while sparing parents for family sustenance.

**Fig. 5:**
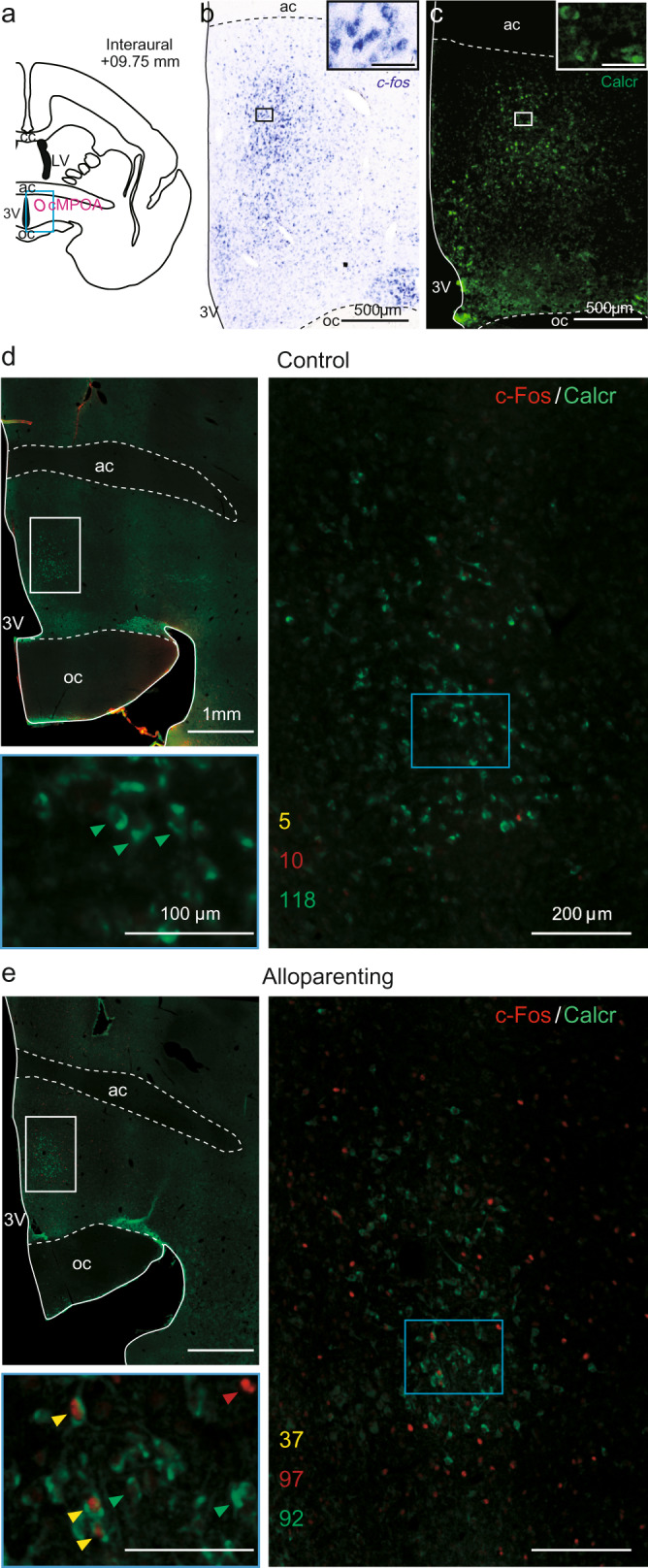
Neurochemical analyses of the marmoset MPOA. **a** The location of photomicrographs (Fig. 5b, c) as a blue rectangle, and the conservative contour of the cMPOA (pink). **b** Distribution of *c-fos* mRNA (blue-purple) in the MPOA of a female older sibling (Charlotte) after alloparenting. Scale bar: 500 μm (main panel) and 50 μm (small panel). **c** Distribution of Calcr (green) in the MPOA of a retired father (Tochan). Scale bar: 500 μm (main panel) and 50 μm (small panel). **d** Double immunohistochemical staining of c-Fos (red) and Calcr (green) in the cMPOA of the control animal (Umi). The numbers of immunoreactive neurons are shown in yellow (double-ir), red (c-Fos-ir), and green (Calcr-ir). An area framed in blue was magnified. Green arrowheads indicate Calcr-ir neurons. **e** Double immunohistochemical staining of c-Fos (red) and Calcr (green) in the cMPOA of an alloparenting animal (of which name Slightly). The numbers of immunoreactive neurons are shown in yellow (double-ir), red (c-Fos-ir), and green (Calcr-ir). An area framed in blue was magnified. Yellow, red, and green arrowheads indicate double-ir, c-Fos-ir, and Calcr-ir neurons, respectively.

As we have recently identified Calcr-expressing neurons in the cMPOA as the most significantly activated and indispensable for parental and alloparental care in mice^13,23,24^, we sought the marmoset counterpart area of the mouse cMPOA. First, in the whole marmoset MPOA, Calcr immunoreactivity and *c-Fos* mRNA (as a readout of transcriptional activation of neurons) were separately visualized in serial sections of older siblings that experienced infant care for 25 min (Fig. 5a–c), elucidating the similar distribution patterns in the central-to-dorsal part of the posterior MPOA in marmosets. Next, Calcr and c-Fos immunoreactivities were simultaneously visualized in infant exposed and non-exposed marmoset caregivers (Fig. 5d, e) and counted the number of colocalizations in the marmoset cMPOA. The double immunohistochemical analysis revealed that 28.7% of Calcr-ir neurons were also c-Fos-ir in the alloparenting animal, while only 4.1% of Calcr-ir neurons were c-Fos-ir in the control animal. These data suggest that molecularly-defined MPOA neurons are involved in infant caregiving in mice and marmosets. Based on these, we defined this marmoset MPOA subregion as being homologous to the mouse cMPOA in terms of neurochemistry, and hereafter call this subregion as the marmoset cMPOA for simplicity (see also the Discussion).

### Functional neuroanatomy of the marmoset MPOA: experimental design

To examine the effects of functional suppression of the above-mentioned marmoset cMPOA on the performance of infant retrieval assays, older siblings were first subjected to adeno-associated viral-vector mediated expression of inducible tetanus toxin (AAV-Tet-ON) by doxycycline (DOX), aiming to reversibly inhibit cMPOA neuron firing as performed in laboratory mice. However, this method yielded insufficient effects in the present study (see Supplementary Figs. 6 and 7 for results and the Method), as well as chemogenetic strategies we have tested separately. To maximally utilize the animals, for the eight animals which showed intact infant carrying after the Tet-ON experiment (Supplementary Data 2, Supplementary Figs. 6–7), we immediately performed a second surgical experiment that utilized targeted excitotoxic lesions with n-methyl-d-aspartic acid (NMDA) (Supplementary Data 2 and Supplementary Figs. 6–7 for manipulation details). This method has been confirmed to specifically overexcite and eliminate the neurons expressing NMDA receptors, without affecting fibers of passage^58,59^. Brain lesion studies have provided numerous pivotal findings on the neural basis of behaviors, particularly in primates. Moreover, the excitotoxic lesion method enabled fine anatomical analyses for the affected subregion (see below), or voxel-based lesion-behavior mapping^60^. The major shortfall of the NMDA lesion method was the non-specific deleterious effects caused by brain damage in general, and should be counterbalanced by the similar-sized lesions in another brain area^60,61^. Thus we employed the NMDA lesions at the posterior septum as a control experiment. The reason for choosing the septum is twofold; first, although septal lesions have been demonstrated to disorganize particular maternal responses in rats and mice, they do not interfere with maternal motivation per se^62–64^. Second, to target the cMPOA, the cannula inevitably passed through the posterior septum, and this minor injury of the septum could be the cause of any observed behavioral change. Considering these features, NMDA lesions of the posterior septum serve as the most stringent control group for counterbalancing the side effects of the NMDA lesions of the cMPOA, compared to the much milder sham lesions that would have been produced if saline or NMLA, the chimeric isomer of NMDA without neurotoxic effects, were to be injected into cMPOA. We also included non-surgical controls to visualize the effects of any experimental manipulations on caregiving parameters (Supplementary Fig. 8).

**Fig. 6:**
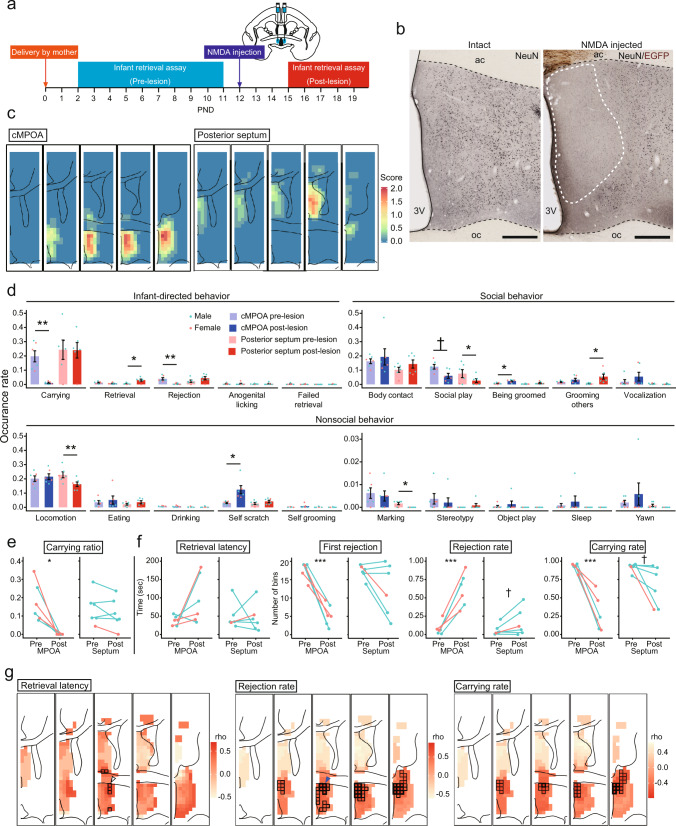
NMDA lesions of the cMPOA but not the posterior septum impair parental tolerance. **a** An experimental timeline. After delivery of a mother, the infant retrieval assay was conducted over 10 days starting from PND 2. On PND 12, NMDA was bilaterally injected into the cMPOA. Then the infant retrieval assay was conducted again from PND 15 to PND 19. **b** An example of the NMDA lesions. Left panel: NeuN immunoreactivity of intact marmoset (Mogol) at the level of the cMPOA. Right panel: lesions in the MPOA in the NMDA injected marmoset (James). An area with neuronal loss was indicated as the dashed-white line. Immunostaining with NeuN (black) and TeNT.EGFP (brown). Expression of TeNT.EGFP before making NMDA lesions did not have behavioral effects (see Supplementary Fig. 7a). Scale bars: 500 μm. **c** A lesion map shows the distribution of bilateral lesions for each group. Scores are calculated as no bilateral lesion (0 points), a partial bilateral lesion (1 point), and a complete bilateral lesion (2 points) for each 250 × 250 μm grid, then averaged among subjects (cMPOA; *n* = 6 (3 males and 3 females), posterior septum; *n* = 6 (5 males and 1 female). The posterior diffusion found in the fifth MPOA panel has been observed as in mice, possibly due to the abundant longitudinal passing fibers in this area^13^. **d** Mean ± SEM occurrence rates of various behaviors in each group before and after the lesion. Upper left panels: infant-directed behaviors. Upper right panels: other social behaviors. Lower panels: non-social behaviors. Blue-green dots: male, pink dots: female. (Welch’s paired *t*-test, cMPOA; *n* = 6, posterior septum; *n* = 6, **p < 0.01, *p < 0.05, ^†^*p* < 0.10) **e** Changes in carrying ratio observed by scan sampling of carrying behavior in the family cage before and after the NMDA injection. Left panel: cMPOA-lesioned group. Right panel: posterior septum-lesioned group. Blue-green: male, pink: female. (Welch’s paired *t*-test, *n* = 6 per group, *p < 0.05). **f** The results of the infant retrieval assay before and after the lesion. Left panels: cMPOA-lesioned group. Right panels: posterior septum-lesioned group. Blue-green: male, pink: female. (Welch’s paired *t*-test, *n* = 6 per group, ***p < 0.001, ^†^*p* < 0.10). **g** Grid-based correlations between the degree of cell loss relative to the same grid in the intact animal and the behavioral performance after surgery. Each grid is 250 μm × 250 μm. The darker grids denote a higher correlation between the behavior and damage. Grids with black frame: *p* < 0.05; grids with bold black frame: *p* < 0.01. (Spearman’s rank correlation coefficients, *n* = 12 including both the cMPOA and posterior septum groups).

The older sibling marmosets were examined for their infant care during PND 2 to 11, given a surgery of NMDA injections at the cMPOA or the posterior septum (*n* = 6 each) at PND 12, and subjected to post-lesion infant retrieval assays during PND 15–20, and their brains were then analyzed histologically (Fig. 6a, Supplementary Figs. 9–13). The cell loss in each grid covering the whole MPOA, posterior septum, and the anterior cingulate cortex (A24a-d), that was situated along the cannula track, was quantified and presented as the mean bilateral cell-loss score for each experimental group (Fig. 6b, c, Supplementary Figs. 9–12). This histological evaluation confirmed the proper targeting of the cMPOA or the posterior septum. Among the six cMPOA lesioned subjects, the average lesioned area covering the conservative contour of the bilateral cMPOA (as depicted in red contour in Supplementary Fig. 13, the right and left hemispheres were measured separately) was 68% (range: 37–94%) (Supplementary Table 1).

### Targeted bilateral lesions at the marmoset cMPOA selectively impaired tolerance to infant carrying in alloparents

A comparison of pre-and post-NMDA injection periods did not reveal any changes in body weight or general health in either of the alloparent groups. Nor did these lesion surgeries induce any gross behavioral changes, such as increased agonistic encounters with other family members; thus all of these older siblings were successfully reunited with the family after two days of recovery from the surgery. The lesion effects on general home-cage behaviors were assessed by the scan sampling and the 20 min family observation. For general nonsocial behaviors, total locomotion and scent marking decreased after the septal lesions, but did not change after the cMPOA lesions (Fig. 6d, lower). The increased incidences of being-groomed and self-scratching after the cMPOA lesions were mostly targeted toward the silk sutures that were applied to the initial four of the cMPOA lesion marmosets, suggesting a possible experimental artifact. Much less being-groomed was observed in the remaining cMPOA or septal marmosets that received stainless-steel surgical clips instead of silk sutures. The other behavioral measures were unaltered in both groups.

For infant-directed behaviors, the cMPOA lesions markedly decreased infant carrying in both focal and scan family observations (Fig. 6d upper left for 20 min observation, and Fig. 6e for scan sampling) and concomitantly the absolute number of rejection (not rejection rate) was also decreased since rejection occurs only during infant carrying. Other infant-directed behaviors, such as the absolute value of retrieval incidences, anogenital licking, and failed retrieval, occurred rarely in the 20 min observation period, and did not appear to be reduced significantly. The posterior septum lesions did not alter infant carrying in both observations (Fig. 6d, e) but did increase infant retrieval. For other social behaviors (Fig. 6d), the septum lesions decreased social play and increased grooming toward others, while the cMPOA lesions marginally decreased social play only (*p* = 0.0587).

The infant retrieval assays confirmed a robust decrease in infant carrying after the cMPOA lesions (Fig. 6f, see also Supplementary Fig. 6 for raw data for each animal). The cMPOA lesioned older siblings mostly retrieved the infant (Fig. 6f, no significant alteration of retrieval latency), but started rejection uniformly earlier than the pre-surgery period (Fig. 6f, first rejection latency), and exhibited an increased rejection rate and a decreased carrying rate. The posterior septum lesions marginally increased the rejection rate (*p* = 0.0935) and decreased the carrying rate in this assay (*p* = 0.0674). The cMPOA and the posterior septum lesions were further compared with PND-matched intact siblings as a non-surgical control group by infant retrieval assays (Supplementary Fig. 8). The cMPOA lesions significantly increased rejection rate and decreased carrying rate in the post-lesion period compared to both the posterior septum and the intact control groups, while the septal lesions did not alter any parameters compared to the control. All these data consistently indicated the robust and specific role of the cMPOA in infant tolerance and total infant carrying. In contrast, the effect of the posterior septal lesions on infant care was relatively mild and variable, and might be partly secondary to the reduced locomotor activities observed in this group.

Two confounding factors arising from the complex experimental history of each subject animal should be considered here; one is that four out of the six MPOA lesioned marmosets received bilateral AAV injections, and the other two received a unilateral AAV injection along with a contralateral NMDA injection as the pretreatment prior to the bilateral NMDA lesions of the cMPOA (Supplementary Fig. 6, the second row); another is that two of the septal lesioned marmosets received a unilateral cMPOA lesion in combination with the AAV injection on the contralateral side, while the remaining four did not receive any pretreatment (Supplementary Fig. 6, third and fourth rows, Supplementary Data 2). To evaluate the effects of these pretreatments, we presented all the raw data of infant retrieval assays (Supplementary Fig. 6) and summarized the result accordingly (Supplementary Fig. 7). Although the limited sample size of each subgroup precluded formal statistical analyses, none of these pretreatments appeared to influence infant-directed behaviors by themselves, or in combination with the second surgery. For example, septal lesioned marmosets with no pretreatment (lr-NMDA (septum) group in Supplementary Figs. 6 and 7b) did not perform any better than those that additionally received unilateral AAV and contralateral NMDA injections into the cMPOA during the pretreatment period (l-AAV/r-NMDA (MPOA) then lr-NMDA (septum) group in Supplementary Figs. 6 and 7b). Therefore, these different pretreatments did not seem to grossly affect the results. It is also worth pointing out that the four marmosets that were pretreated with bilateral AAV injections into the MPOA showed normal alloparental behavior, indicating that ‘sham’ lesions of the MPOA do not impact marmoset alloparental behavior.

In addition, due to the limited availability of experimental subjects, we could not counterbalance the sex of subjects for posterior septal lesions (only one was female and five were males), while three were in the cMPOA lesion group. Thus we showed the sex by color in Fig. 6d–f, and did not find obvious sex differences in the pre-and post-surgical infant care behaviors.

### Grid-based lesion-behavior mapping revealed the specific MPOA subregions required for each dimension of alloparenting styles

Finally, we utilized a grid-based lesion-behavior mapping technique to delineate the responsible brain subregions within the analyzed brain area (the whole MPOA, posterior septum, the adjacent bed nuclei of stria terminalis, and the cingulate cortex in the five coronal sections in Fig. 6g) for each dimension of alloparenting styles. The cell loss at each grid was scored bilaterally, because the unilateral lesions in the mouse cMPOA affect caregiving behaviors only mildly^13^. Correlation analysis between the resultant cell-loss score and the rejection rate changes between pre- and post-lesion periods (Fig. 6g) demonstrated significant negative effects of lesions in the central-to-dorsal posterior MPOA, which overlapped with the Calcr and c-Fos expression in the Fig. 5e. The subregions associated with the retrieval latency change (the white arrowhead in retrieval latency of Fig. 6g) were located slightly more lateral than those associated with the rejection rate change (the blue arrowhead in rejection rate of Fig. 6g). In addition, the ventral anterior cingulate and the dorsal posterior septum were damaged relatively more in the subjects that showed higher postoperative retrieval latencies, but these did not reach statistical significance (the orange-colored area around the corpus callosum in retrieval latency of Fig. 6g). A separate analysis mapped the lesions that were correlated with a decrease in social play and identified the posteromedial part of the bed nucleus of stria terminalis (Supplementary Fig. 14), which was segregated from the subregions correlated with rejection rate or carrying rate. The identified brain area has been shown to express c-Fos after social play in male rats^65^. Overall, our lesion-behavior mapping analyses revealed the precise brain area where cellular functions were associated with alloparental tolerance at the central-to-dorsal posterior MPOA in common marmosets.

## Discussion

The present study systematically analyzed the family-shared infant care of common marmosets across multiple social settings, and identified caregiver-inherent parenting and alloparenting styles. In particular, the retrieval latency and the rejection rate vary widely across individuals, and are fairly constant within each caregiver across birth and litters, and thus appeared intrinsic to each caregiver. Utilizing these parameters, each caregiver can be plotted onto a two-dimensional map as shown in Fig. 3h. The sensitivity-tolerance dimensions of infant caregiving found in the present study have substantial consistencies with maternal styles reported in Old World monkeys^66–71^, and further extend these into paternal and alloparental caregiving. In humans, Bowlby summarized the parameters as the maternal sensitivity in responding to her baby’s signals, and the quantity and quality of interaction between mother and baby^72,73^. Based on Baumrind^74^, McCoby^75^ proposed two parenting dimensions responsiveness and demandingness/control, and these are the best-accepted descriptors of parental characteristics among researchers^76,77^. Of course, psychological constructs for humans are very complex and are not completely comparable to the simpler behavioral parameters observed in marmosets. Still, from the ethological point of view, there is at least some conceptual overlap of infant retrieval latency with human parental responsiveness, which includes the measure of parental response to signs of infant distress. Parental control or demandingness in humans has been discussed in the context of discipline^78^, and includes corporal punishment such as spanking. Thus it could also share some features with the physical rejection of the infant in marmosets. Detailed discussion on this issue is avoided here, but it is an intriguing viewpoint with regards to the evolution of primate parenting and deserves further investigation.

This study next attempted to identify the brain substrate of parenting styles. To do this invasive experiment, this study focused on alloparents (older siblings), and did not examine the involvement of the cMPOA in postpartum maternal or paternal care. We took this approach because utilizing the parents for invasive experiments would have inevitably destroyed the whole family and was therefore avoided. Moreover, the accumulating rodent evidence strongly suggests a common neural basis for maternal, paternal, and allomaternal care that includes the cMPOA^5,13,23,24,56^ (but in ref. ^57^). However, as a major limitation of this study, we could not directly manipulate Calcr-expressing neurons but relied on neurotoxic lesions for functional analyses. To overcome the identified shortfalls of permanent lesion methods^61^, we utilized multiple dissociation logic^60^ by employing many behavioral measurements including non-social and social behaviors in different social contexts, as well as the similar-sized posterior septal lesions, and non-surgical controls. We also performed the fine-anatomical mapping of the grid-based lesion-behavior correlation analysis (Fig. 6), which could be done most precisely by permanent lesion methods^60^.

Based on these rationales, this study first demonstrated that the Calcr-expressing cMPOA neurons were indeed activated during infant care, as in laboratory mice. Then, ablating the marmoset cMPOA subregion of the MPOA significantly impaired parental tolerance and infant care quantity, without grossly affecting other social and non-social behaviors. Moreover, the robust and specific function of the cMPOA on infant care was shown to be consistent across different social settings, but this was not so for the posterior septum, the latter being consistent with rodent literature^63,64,79^. Therefore, the role of cMPOA subregion in the regulation of alloparental behavior in primates has been demonstrated clearly. Obviously however, this study did not exclude the possibility that non-Calcr-expressing neurons within the cMPOA are actually responsible for alloparental tolerance, instead of Calcr-expressing neurons. Subsequent research should perform pharmacological, genetic, or other cell-type specific manipulations to confirm the functional importance of Calcr-expressing neurons for marmoset parental and alloparental care.

The original acronym of the cMPOA in mice was used to designate the central part of the posterior MPOA as the functionally indispensable subregion for parental behaviors within the MPOA^13^. This study has identified the marmoset counterpart of the mouse cMPOA neurons by using neurochemical markers to delineate the co-expression of Calcr and c-Fos in the central-to-dorsal part of the posterior MPOA. However, spatial localizations of particular neurons may vary along the A-P axis, among individual animals, and across different species, and cannot be precise. And just vaguely calling the area the MPOA is confusing, because the whole MPOA is as large as the whole amygdala and contains at least 12 subregions that are distinctive for neurochemical and functional features^22,80^. Therefore, we propose to switch the acronym of the cMPOA to refer to the Calcr-expressing MPOA subregion, relying more on neurochemical features conserved across mice and marmosets.

A dominant view derived from the rodent literature has proposed that MPOA neurons regulate maternal responsiveness by (1) activating infant acceptance and by (2) depressing infant rejection^5,81–83^. Calcr-expressing cMPOA neurons may be involved in both functions in mice, as their selective silencing drastically delays pup retrieval in virgin and maternal females, and their chemogenetic activation abolishes infanticide of sexually-naïve male mice^24^. In this study, however, the cMPOA lesions in marmosets suppressed alloparental tolerance while leaving alloparental sensitivity or retrieval latency, which can be regarded as appetitive parental acceptance of infants, largely intact. This effect could have occurred because a slightly more lateral location of the responsible MPOA subregion for retrieval latency was not sufficiently damaged by our NMDA lesions. Another possibility is that in marmosets, the approaching component of infant care receives dual facilitations, appetitively by the MPOA^5,84^ and also as an aversive response to loud distress vocalizations by infants, with this facilitation being regulated by another brain area, such as the amygdala^85^. This issue is certainly worth considering in future studies.

Taken together, our study suggests the existence of evolutionary-conserved behavioral and neural mechanisms for infant care behaviors in mammals, and provides a necessary step to dissect the neural basis of primate parenting and alloparenting styles. Of course, subsequent research that selectively manipulates the neuronal activities, the receptor activity by agonism or antagonism, the up-or down-regulation of receptor expression, or by investigating the connectivity of Calcr expressing neurons with other brain regions and with other neuroendocrinological factors such as estrogen and oxytocin^33,43,86,87^ are required to establish the functional role of these neurons in marmoset parental and alloparental behaviors.

## Methods

### Animals and family observations (scan/focal)

All animal experimentation was approved by the Animal Experiment Judging Committee of RIKEN and was conducted in accordance with the Guide for the Care and Use of Laboratory Animals, 8th edition (the National Research Council of the National Academies, 2011, The National Academies Press).

We tested 81 common marmosets from 10 families: 9 fathers, 10 mothers, 43 siblings (26 males and 17 females), and 22 infants (10 males and 12 females). Beads, Cubby, Pon, and Ribbon were examined as siblings and also as parents (three fathers and a mother). A separate non-parent adult male (Umi) was used as a control for c-Fos expression analysis. All of these marmoset subjects were kept at the CBS of RIKEN, Japan. Detailed information about the subjects is shown in Supplementary Data 1. They were housed as a family, ranging from parents and two infants (minimum) to parents, two older siblings, two younger siblings, and two infants (maximum). The average number of infants at one birth was 2.21. When triplets or quadruplets were born, the larger two were kept in the family and the rest was permanently removed from the family on PND 0-2 to be subjected to another experiment. In the Chuk’s family, the mother (Fastener) could nurture only one infant because of the dysfunction of one nipple, so when twins were born, one infant was removed. These removed infants were excluded from data analysis. Infant mortality before PND 30 (indicated as square and curly brackets in Supplementary Data 1) was 12% (10 of 84, excluding those subjected to other experiments right after birth). In the case of Chuck’s family, there was one delivery of a singleton between Birth 1 and Birth 2, but that infant died on PND 0, so data was not obtained for this birth. In Chuck’s family’s Birth 6, all infants died by PND 6, thus this birth was excluded from data for the scan sampling of carrying behavior. Two animals died well after weaning (PND 161 and 208), which did not affect the results of the observations and Experiments in this study.

The home-cage was 43(wide) × 66(height) × 60(deep) cm. Two or three cages were joined through a square hole (9.6 cm wide× 10.5 cm height) on a side panel or a metal mesh tunnel (75 cm wide × 30.5 cm height × 21 cm deep) placed in front of two cages, depending on the number of family members in accordance with the ethical guideline of RIKEN. The cage contained a food tray, a water faucet, two wooden perches, and a metal-mesh loft. Although tactile contact was restricted between families, visual, olfactory, and auditory communication was possible in the colony room. The animals were fed a monkey diet around 11:30 and supplementary diets such as a piece of a sponge cake, dried fruits, and lactobacillus preparation around 16:00. Water was supplied ad libitum. The photoperiod of the colony room was 12 L:12D (light period: 8:00–20:00, dark period: 20:00–8:00). The observations and experiments were conducted in the animals’ home cage between 8:00 and 17:00. All marmosets were well habituated to the presence of the experimenters (KS, SY, and a technical staffs) in the colony room to conduct the observations and experiments.

### Instantaneous scan sampling of the family cage

Instantaneous scan samples of infant carrying behavior in families were performed essentially as described in ref. ^41^; briefly, scans were taken five times a day (9:00, 11:00, 13:00, 15:00, and 17:00) on weekdays until PND 83. Additional samplings were also occasionally made on weekends. The author or a trained observer entered the colony room and recorded the identity of the carrier(s) and the number of infants being carried.

### Continuous family observation

Each family was observed for 20 min observation periods once (minimum) to seven times (maximum) per week until PND 60. Ninety percent of observations (472 out of 526 observations) were conducted in the afternoon (12:00–18:00). The remaining observations occurred either in the morning (8:00–12:00, 31 observations, 6%), or at a time that was not recorded (23 observations, 4%). Before starting the observation, all the family members were lured into two-joined compartments, because it was difficult to observe the behaviors of all the family members on-site if they are distributed in three compartments. Observations were recorded on the check sheets by KS. Each family member’s caretaking behavior, social behavior, and non-social behavior were recorded for 20 min with 30 s bins (Table 1).

### Infant retrieval assay

The dyadic infant-retrieval assays (Fig. 3a), or brief separation-reunion sessions from the infant’s viewpoint, were based on the previous marmoset literature^43,51^ and rodent parental-behavior studies^88–90^. The stress loaded onto the subject animals was minimized by the use of their home cage as the testing arena, although some stress was inevitable by the temporary separation of other family members from the testing area, and a brief period (maximally 10 min) of infant isolation.

This assay was conducted from PND 1 to PND 41. An infant, which was the subject’s own offspring or the subject’s own younger sibling, was presented as the stimulus animal when we returned the infant to its home cage after daily body-weight measurement. If more than two subjects from each family were tested in the same parturition or an infant was a singleton, the infants were separated twice on the same day. The order of the test of multiple family members was counterbalanced. When there were twins in one family, the stimulus animals were alternated. All subjects were acclimatized to the tunnel and the wire-mesh basket without infants before the test. During the test, the joined-home cages were divided into three cages (43 × 66 × 60 cm each) using steel partitions. Two cages were connected by a mesh tunnel (75 × 30 × 21 cm) and used for the test (Fig. 3a). The subject was placed in the middle cage before the start of the test. The other family members were placed in the left cage. During this procedure, the subject and the other family members were gently separated by luring them with a piece of sponge cake to minimize the effect of handling on the subject’s subsequent behavior. Then the stimulus infant was gently taken out of a carrier and placed into the mesh basket (15 cm diameter × 15 cm high), which contained a gauze-covered electric hand warmer (KIR-SE1S, Sanyo, Osaka, Japan), to maintain the body temperature of the infant during separation. The infant in the mesh basket was placed in the right cage. Opening the shutter at the subject cage permitted the subject access to the infant’s cage. The behavior of the subject before retrieval and 600 s after retrieval was directly observed and recorded using two video cameras (HDR-AS100V, Sony, Tokyo, Japan) as well as a directional microphone (MKH 416, Sennheiser, Hanover, Germany) connected to a linear PCM recorder (DR-60DMKII, Tascam, Tokyo, Japan). The audio was recorded at 24-bit and 96 kHz. The time from the opening of the sliding door to the retrieval of the infant, which was defined as when all of the infant’s limbs were in contact with the subject’s body, was recorded as the retrieval latency. Immediately after the successful retrieval, the caregivers’ infant-directed behaviors were coded for 10 min with 30 s bins. The session ended if the subject caregiver did not retrieve the infant for 600 s (or 300 s for the initial 12.6% of this research). Twenty-three (2.2%) and 39 (4.2%) sessions were ended without retrieval for 300 s and 600 s respectively, among 1086 sessions in total. For consistency, the data in Fig. 3d–n were calculated by assigning the sessions that had longer than 300 s retrieval latency as 300 s. The data in Fig. 6f were calculated as it is, as all the sessions used the limit of 600 s.

On-site observation coded caregivers’ behavioral repertoire is listed in Table 1, except for body contact, social play, being groomed, and grooming others, because these behaviors will not occur in the absence of other family members in test cages. The results of these on-site observatory records were used in this study. The detailed behavioral analyses of infant-caregiver interactions including vocal communication were performed off-site with the video and audio recordings and will be presented in another paper.

### Food transfer assay

The food transfer tests were performed as previously described in ref. ^42,43^, and were conducted beginning on PND 68–90 and continuing one or two sessions/week for six weeks in the animals’ home cage between 13:00 and 17:00. Water was available ad libitum. Until just before the experiments, the monkey diet was available ad libitum. For the experiments, one caregiver and one juvenile were placed in one cage and separated from the other family using a partition (Fig. 4a). The test session started with the presentation of a familiar preferred food (steamed sweet potatoes chopped into 1 cm cubes or puffed rice cereals) that was placed about 10 cm away from the front of the cage on a white acrylic board (29.5 cm wide × 24.0 cm deep), which only the caregiver could reach. The distance of food items from the front of the cage was adjusted on-site for each caregiver. In the initial half (47.0%, 455 out of 969 sessions) of the experiments, 5 cubes of steamed sweet potatoes were used as the food and the session was 5 min. Then we changed the experimental design to use rice cereals for 15 min sessions (51.6%, 500 out of 969). The 1.4% of sessions (14 out of 969) in between were preparatory sessions for testing different foods and different session times, ranging from 500 s to 1448 s.

At the start of the session, 5 pieces of food items were continuously presented by replenishing them. Each caregiver was tested once a day, 1 or 2 times a week depending on the period of execution in the study. Each offspring was used one to two times for a test day. Therefore, the total number of sessions for each caregiver was 6 to 12. The order of caregiver and offspring combination was counterbalanced.

The behavior of both caregiver and offspring during each test session was videotaped and analyzed later. The total number of cubes of potatoes picked up by the caregiver was counted. In addition, the number of times each of the following behaviors occurred was also recorded. Interest: An offspring approached the caregiver within a distance of 10 cm and looked toward the food item while the caregiver had the food item in his/her hand. Begging: An offspring vocalized while exhibiting interest. The bout of interest and begging was defined to have ended when the distance between the caregiver and the offspring became more than 10 cm or when the food item had been completely eaten or dropped. Refusal: A caregiver moved away from, turned its back on, emitted threat vocalization to, or pushed away an offspring showing interest in the food item. Transfer: An offspring licked or took a bite from the food item being held by a caregiver, or took the whole food item. The transfer was necessarily preceded by a bout of interest, but not necessarily by begging. Food transfer could occur after a caregiver showed refusal. These behavioral categories approximated those used by^91^.

### Stereotaxic surgery for viral vector injections and excitotoxic NMDA lesions

In total, 20 older siblings received AAV-mediated and/or NMDA lesion interventions. The dates for these interventions are summarized in Supplementary Data 2 for individual animals. The points of removal of any family members were also shown in Supplementary Data 2 and Fig. 1c (bars).

Sixteen marmosets received viral vector injections before the mother’s next delivery in the family group (Supplementary Data 2). Two of them were excluded from the analysis; one (Cubby) was removed because the experiment was terminated due to infant mortality, and another (George) could not reunite with the original family, because the separation period was exceptionally prolonged to two weeks due to the re-opening of the surgical sutures; therefore, a total of 14 subjects were included. Viral vectors used were 1:1 mixture of AAV2-CMV-rtTAV16 -WPRE-SV40pA and AAV2-TRE-eTeNT.Flag -WPRE-SV40pA (titers: 1.15E + 13 and 1.00E + 13 particles/ml respectively, *n* = 3) or AAV-DJ/8-TRE-EGFP.eTENT.PEST-WPRE-pA and AAV-DJ/8-CMV-rtTAV16-WPRE-pA (titers: 2.4E + 13 and 4.6E + 13 particles/ml respectively, *n* = 7 for bilateral virus injection, and *n* = 4 for unilateral virus and NMDA injection). For bilateral virus injections, on the day of stereotaxic surgery, animals were deeply anesthetized first with an intramuscular injection of a mixture of xylazine (2.4 mg/kg) and ketamine (30 mg/kg), then with inhalation of isoflurane (1 to 3% in air); then the viral solution was injected stereotaxically into the bilateral MPOA through a pulled glass capillary (tip diameter 20–50 μm) by oil pressure under sterile conditions. The stereotaxic coordinates based on the interaural zero reference point were taken from the marmoset brain atlas^92^: A + 10.3 mm, L ± 0.6 mm, V + 6.8 mm. The injection volume was 80–250 nl/side.

For unilateral virus and NMDA injection, the virus solution was injected into the left side of MPOA in the same manner as described above. Then 500 nl of the excitotoxic amino acid, N-methyl-D-aspartic acid (NMDA; M3262, Sigma-Aldrich, St. Louis, MO, USA; 20 mg/ml saline) was injected into the right side of MPOA. This procedure was applied to PakChee, Sage, Ataro, and Setaro, with the expectation that it would have a disruptive effect on alloparental behavior in conjunction with the TeNT effect on the virus-injected side of MPOA. However, this procedure did not have a behavioral effect. Neither did the bilateral virus injection have a behavioral effect (Supplementary Figs. 7b, 9–12). After surgery, the wound was closed by silk suture or surgical clips and the subjects were treated with analgesics and antibiotics. During the 2-day recovery period after surgery, they were separated in one of the home cages but allowed to have visual/olfactory access to family members through the mesh door in the tunnel. After the mother’s delivery, they served as subjects for behavioral observations and the infant retrieval assay. They received daily oral administration of the doxycycline (DOX; D9891, Sigma-Aldrich, St. Louis, MO, USA; 20 mg/kg) after the infant retrieval assay from PND 7 or PND 10 (depending on the subject, see Supplementary Fig. 6), to express the tetanus toxin in the injected area. As a result, however, we did not obtain significant changes in the infant retrieval assay before and after the administration of the DOX (Supplementary Fig. 7a). To maximally utilize the animals, six were perfused 30 min after the last infant exposure for histological analysis of caregiving-induced *c-Fos* mRNA, while the remaining eight subjects were used for the lesion experiment described below.

Thirteen marmosets received a bilateral injection of NMDA aiming at the cMPOA or posterior septum on PND 12 or 13 of newborns (Supplementary Data 2; including eight AAV-injected animals, six for the cMPOA, two for the posterior septal lesions). One (Rin) of them was a pilot experiment and excluded from the analysis, so the number of subjects was *n* = 6 for both cMPOA and posterior septum groups. Prior to the lesions, these subjects continuously cohabited with their parents and experienced caretaking of newborns in a previous delivery of the mother. Before surgery, family observations were conducted daily from PND 0 to 11. Also, the infant retrieval assay was conducted daily from PND 2 to PND 11. These data served as a within-subject control. On the day of the stereotaxic surgery, animals were deeply anesthetized first with an intramuscular injection of a mixture of xylazine (2.4 mg/kg) and ketamine (30 mg/kg), then with inhalation of isoflurane (1 to 3% in air); then The NMDA solution at 20 mg/ml in saline was bilaterally injected into the cMPOA or posterior septum through a pulled glass capillary (tip diameter 20–50 μm) by oil pressure under sterile conditions. The stereotaxic coordinates based on the interaural zero reference point were taken from the marmoset brain atlas^92^: for the cMPOA, A + 10.3 mm, L ± 0.6 mm, V + 6.8 mm; for the posterior septum, A + 10.3 mm, L ± 0.6 mm, V + 10.5 mm. The injection volume was 500 nl/side both for the cMPOA and septum. After surgery, the wound was closed by silk suture or surgical clips and the marmosets were allowed a 2-day recovery period. During this period, they were separated in one of the home cages but allowed to have visual/olfactory access to family members through the mesh door in the tunnel. Then they were subjected to behavioral experiments again for 5 days.

After the experiment, brain sections were prepared and the injection site was confirmed with immunohistochemical staining with anti-NeuN antibody (1:1000, MAB377, Millipore). Brain sections of virus-injected animals were also stained with anti-Flag (1:250, F1804, Sigma-Aldrich, St. Louis, MO, USA) or anti-GFP (1:10000, Code No. 598, MBL, Tokyo, Japan) antibodies to confirm expression of eTeNT although it did not have behavioral effects (Fig. 6b, black: NeuN, brown: GFP). Then analysis for NMDA-induced neuronal loss was performed. The boundary of the bilateral lesion area (>70% of neuronal loss for each side) was drawn (Supplementary Figs. 9–10; the boundary of the lesion area in each side, Supplementary Figs. 11–12; bilateral lesion area colored in dark blue). Then 35 (dorsoventral) ×10 (mediolateral) of 250 vm × 250 μm grids were overlayed and scored as 0 (no inclusion of bilateral lesion area), 1 (partial inclusion of bilateral lesion area), or 2 (complete inclusion of bilateral lesion area) for each grid in the whole MPOA, septum, and the anterior cingulate cortex appeared in the five serial sections shown in the Fig. 6c. The percentage of the cMPOA damaged on each side for the cMPOA lesion group was also calculated (Supplementary Table 1 and Supplementary Fig. 13).

One caveat is that we did not perform an NMLA control, a chimeric isomer of the NMDA. However, the fact that NMLA does not cause any neuronal loss has been well established in rodent studies, and the infusion of a liquid into the cMPOA through the glass pipette did not affect caregiving behavior as evidenced by the AAV injection experiment.

Another caveat of this experiment is that all six animals in the cMPOA lesioned group were subjected to prior AAV injection, while only two animals in the posterior septal NMDA lesioned group received prior AAV injections in cMPOA. Considering the within-subject control in the cMPOA group and the AAV-treated septal lesion animals (Setaro and Ataro), the prior AAV treatment was unlikely to affect the caregiving performance.

Lastly, we have changed the method of surgical wound closure from silk sutures to surgical clips during the course of this experiment; while 4 of 6 cMPOA lesioned animals received silk sutures, all of the rest of the lesioned animals received surgical clips. We noticed that the silk suture received much more self-scratch and grooming from the other family members. This fact may have caused the increased being-groomed and self-scratch targeted at the suture sites of cMPOA lesioned animals (Fig. 6d).

### Preparation of brain sections

For activation mapping using *c-fos* mRNA or c-Fos protein expression shown in Fig. 5, animals received an infant exposure on the day of perfusion. The subject caregiver was gently lured into a separate cage from family members 2 to 3 days before the infant exposure. On the test day, the experimenter introduced an infant from a front cage-door. Then the subject was allowed to interact with the infant for 25 min (*c-fos* in situ hybridization) or 30 min (c-Fos immunohistochemistry). Each subject’s behaviors were recorded by an experimenter on-site and also by the video camera and the directional microphone. The subject was then anesthetized immediately after the observation (for in situ hybridization) or 90 min after the observation (for immunohistochemistry) (see also Supplementary Data 2).

Animals were deeply anesthetized first with intramuscular injection of a mixture of xylazine (2.4 mg/kg) and ketamine (30 mg/kg) and then with sodium pentobarbital (80 mg/kg, i.p.). Then animals were perfused transcardially with 0.1 M phosphate buffer (PB, pH 7.4) and then 4% (w/v) paraformaldehyde (PFA) in 0.1 M PB. The brains were removed, immersed in the same fixative at 4 °C overnight, followed by cryoprotection in the series of 20 and 30% (w/v) sucrose in PBS until it sank (typically 2 to 3 days each), frozen by being buried in powder dry ice, and stored at −80 °C until sectioning. Brains were sectioned on a freezing microtome at 40 μm according to the marmoset brain atlas^92^ Every 5th section from the serial sections was processed for immunohistochemistry (IHC) or in situ hybridization (ISH) combined with IHC. The remaining sections were used to confirm the reproducibility of antibodies and riboprobes, using at least 2 samples for each molecule. Details of the subject animals are described in Supplementary Data 2.

### Immunohistochemistry and in situ hybridization

Riboprobes for in situ hybridization (ISH) were prepared using the following sequences: nucleotides 521 – 1133 of *c-fos* mRNA (XM_002754121). The mouse cDNA was amplified using PCR with the sense primer (5′-CTGTCTCCAGTGCCAACTTCA-3′) and antisense primer (5′-CTCTTGACAGGCTCCACTGA-3′) containing an artificially introduced SP6 promoter at its 5′ end (5′-ATTTAGGTGACACTATAGAA -3′). The antisense probes were transcribed by SP6 RNA polymerase (P1085; Promega) in the presence of digoxigenin-labelled UTP (Dig labelling mix; Roche Diagnostics, Basel, Switzerland), and precipitation with LiCl with ethanol following a protocol as described in ref. ^13^.

The brain sections were processed for ISH following a standard procedure as described in ref. ^13^ with some modifications. Briefly, the sections were washed with PBS containing 0.1% Tween-20 (PBT), and postfixed with 4% PFA in PBS for 10 min at room temperature. Then they were immersed in methanol containing 0.3% H_2_O_2_ for 15 min, followed by acetylation with 0.25% acetic anhydride in 0.1 M triethanolamine. The hybridization solution contained 50% of deionized formamide, 5× standard saline citrate (SSC, pH 7.0), 5 mM ethylene-diaminetetraacetic acid (pH 8.0), 0.2 mg/ml of yeast tRNA, 0.2% Tween-20, 0.2% sodium dodecyl sulfate, 10% dextran sulfate, and 0.1 mg/ml of heparin. The sections were prehybridized at 58 °C in the mixture of the hybridization solution and PBT (1:1) for 30 min, immersed in the hybridization solution for 30 min; then hybridized with the riboprobes (1 μg/ml) at 58 °C for 16 h. After hybridization, the sections were washed twice with 2× SSC containing 50% deionized formamide at 58 °C for 10 min, incubated with RNAse A solution (20 μg/ml) at 37 °C for 30 min, and rinsed twice in 2× SSC and 0.2× SSC at 37 °C (10 min each). The sections were incubated in a peroxidase-conjugated anti-digoxigenin antiserum (1:10000, Roche Diagnostics) and 5-bromo-4-chloro-3-indolyl phosphate/nitroblue tetrazolium (BCIP/NBT solution kit; Nacalai Tesque, Kyoto, Japan) to visualize the labelling. Subsequently, they were washed with PBS and then mounted on gelatin-coated slides using Softmount (199-11311, FUJIFILM Wako Pure Chemical Corporation).

Immunohistochemistry (IHC) on free-floating sections was performed essentially as described in ref. ^13^. Antigen retrieval was performed with 0.01 M citrate buffer (pH 6) in 10% glycerol at 85 °C for 35 min then room temperature for 30 min, after 1st wash with PBS containing 0.2% Triton-100 (PBST) for 3 h. The sections were washed with PBST, incubated with 0.3% H_2_O_2_ in methanol for 5 min, washed with PBST, blocked with 0.8% Block Ace (Dainihon-Seiyaku, Osaka, Japan), and 10% normal goat serum in PBST. The sections were incubated at 4 °C three overnight with rabbit primary antibodies against Calcr (1:250, AHP635, Bio-Rad) or c-Fos (1:5000, sc-52, Santa Cruz Biotechnology, Inc., Dallas, TX, USA) (note: the staining results were notably varied by the c-Fos antibody lot numbers). The following morning the sections were washed and incubated with biotin-conjugated horse anti-rabbit secondary antibody (1:2000, BA-1100, Vector Laboratories, Inc., Burlingame, CA, USA) for 2 h and then in ABC peroxidase reagent (Vectastain ABC Elite kit; Vector Laboratories) for 1 h according to the manufacturer’s instructions. The fluorescent signals were detected using tyramide signal amplification as described in ref. ^93^. The sections were immersed in 0.1 M boric buffer (pH 8.5) containing 4 μM Alexa488 or 568-labeled tyramide, 4% dextran sulfate, 0.05 mg/ml iodophenol, and 0.003% H_2_O_2_ for 30 min. For c-Fos and Calcr double labeling, following the first staining using c-Fos antibody, the sections were processed similarly for the second staining procedure using an anti-Calcr antibody. Subsequently, they were washed with PBS and then mounted on gelatin-coated slides using a mounting medium (Vectashield; Vector Laboratories).

### Photomicrograph analyses

Photomicrographs were taken using an inverted microscope (KEYENCE BZ-X700, Osaka, Japan) with a 10–40× objective. The contrast and brightness of the all photographs were adjusted only linearly and uniformly for all the micrographs used in one experiment, using software (Image J^94^ or BZ-X Analyzer (KEYENCE BZ-X700, Osaka, Japan)).

The quantification of the distribution of c-Fos-ir and Calcr-ir neurons was manually performed on the Image J within the right panels of Fig. 5d, e (Interaural+9.8, measured area was 1136 μm×760 μm).

### Statistics and reproducibility

Statistical analyses were conducted using software R^95^ and SPSS (IBM, Armonk, NY, USA).

Changes in carrying rate through postnatal weeks were fitted to the logistic curve using the glm function with a binomial distribution on the R software. Other behavioral data were analyzed with Welch’s paired *t*-test, a general linear model, a linear mixed model (LMM, a Gaussian distribution with an identity link function), or a generalized linear mixed model (GLMM, a Poisson distribution with a log link function or a binomial distribution with a logit link function) as specified in each figure. The lme4 package was used for LMM/GLMM. The family, the subject nested in the family, and the infant nested in the family were set as random effects as necessary to control pseudo-replication. Postnatal weeks, the number of siblings, and the number of infants carried were set as a categorical fixed effect into the model to conduct post-hoc paired comparisons. Significant effects in the model were extracted using the step function in the lmerTest package, then multiple comparisons were conducted using the emmeans package with Holm adjustment. Statistical significance was indicated with asterisks and horizontal lines, or lowercase letters; that is, if two variables contain the same alphabet (eg. ab and bc), the difference between these two variables is not statistically significant.

The relationship between the damaged area and the performance during the infant retrieval assay in the lesioned marmosets was examined with the Spearman’s rank correlation coefficients for each 250 × 250 μm grid. Significant correlations that indicated a negative relationship with behavioral performance were illustrated as positive correlations for retrieval latency and rejection rate, and negative correlations for carrying rate.

We used 81 common marmosets from 10 families: 9 fathers, 10 mothers, 43 siblings (26 males and 17 females), and 22 infants (10 males and 12 females) in total. Sample sizes were 7366 scan samples from 29 births of 9 families for Fig. 1, 526 observations from 30 births in 9 families including 48 animals for Fig. 2, 815 trials using 46 intact caregivers in 30 births of 9 families for Fig. 3, 969 sessions using 53 intact caregivers in 35 births of 10 families for Fig. 4, and 12 subjects (6 each for cMPOA and posterior septum lesion groups) for Fig. 6.

### Reporting summary

Further information on research design is available in the Nature Research Reporting Summary linked to this article.

## Supplementary information

Supplementary Information

Description of Additional Supplementary Files

Supplementary Data 1

Supplementary Data 2

Supplementary Data 3

Supplementary Movie 1

Supplementary Movie 2

Supplementary Movie 3

Supplementary Movie 4

Reporting Summary

## Data Availability

The datasets generated during and/or analyzed during the current study are available from the corresponding author on reasonable request. All source data underlying the graphs and charts presented in the main figures are available in Supplementary Data 3.
