# Supplementary material for: A calcitonin receptor-expressing subregion of the medial preoptic area is involved in alloparental tolerance in common marmosets
Source: Commun Biol. 2022 Nov 21;5:1243. doi: 10.1038/s42003-022-04166-2 (PMC9678893; doi:10.1038/s42003-022-04166-2)
Supplement: Supplementary file 1 — Supplementary Information [file 42003_2022_4166_MOESM1_ESM.pdf]

Supplementary Information:
A calcitonin receptor-expressing subregion of the medial preoptic area is involved in alloparental tolerance in common marmosets

Kazutaka Shinozuka[1], Saori Yano-Nashimoto[1,2], Chihiro Yoshihara[1], Kenichi Tokita[1,3], Takuma Kurachi[1], Ryosuke Matsui[4], Dai Watanabe[4], Ken-ichi Inoue[5], Masahiko Takada[5], Keiko Moriya-Ito[6], Hironobu Tokuno[6], Michael Numan[7], Atsuko Saito[8*], Kumi O. Kuroda[1*]

1 Laboratory for Affiliative Social Behavior, RIKEN Center for Brain Science, Saitama, Japan

2 Laboratory of Physiology, Department of Basic Veterinary Sciences, Graduate School of Veterinary Medicine, Hokkaido University, Hokkaido, Japan

3 School of Law, Senshu University, Kanagawa, Japan

4 Department of Biological Sciences, Kyoto University, Kyoto, Japan

5 Systems Neuroscience Section, Center for the Evolutionary Origins of Human Behavior, Kyoto University, Inuyama, Aichi, Japan

6 Department of Brain & Neurosciences, Tokyo Metropolitan Institute of Medical Science, Tokyo, Japan

7 Department of Psychology, University of New Mexico, Albuquerque, NM, USA

8 Department of Psychology, Faculty of Human Sciences, Sophia University, Tokyo, Japan

***Corresponding author:**

Kumi O. Kuroda

Laboratory for Affiliative Social Behavior, RIKEN Center for Brain Science

Hirosawa 2-1, Wakoshi, Saitama 351-0198, Japan

E-mail: kumi.kuroda@a.riken.jp; Tel: +81-48-467-7556, Fax: +81-48-467-6853

Atsuko Saito

Department of Psychology, Faculty of Human Sciences, Sophia University,

Kioicho 7-1, Chiyoda-ku, Tokyo 102-8554, Japan

E-mail: atsaito@sophia.ac.jp

Supplementary Table 1 Rate of lesions in cMPOA.

Values indicated % area of lesions (defined as > 70% of neuronal loss) in cMPOA for each side in each section among subjects in cMPOA lesion group.

	Section 1		Section 2		Section 3		Overall
	L	R	L	R	L	R	
Eugenie	98.41	0.00	100.00	73.93	100.00	66.67	73.17
Henry	100.00	69.67	100.00	100.00	100.00	94.77	94.07
James	0.00	0.00	82.33	100.00	59.48	100.00	56.97
PakChee	0.00	0.00	46.81	25.73	90.85	61.44	37.47
Richard	85.96	93.25	100.00	100.00	76.14	100.00	92.56
Sage	72.72	0.00	20.07	100.00	32.03	100.00	54.14

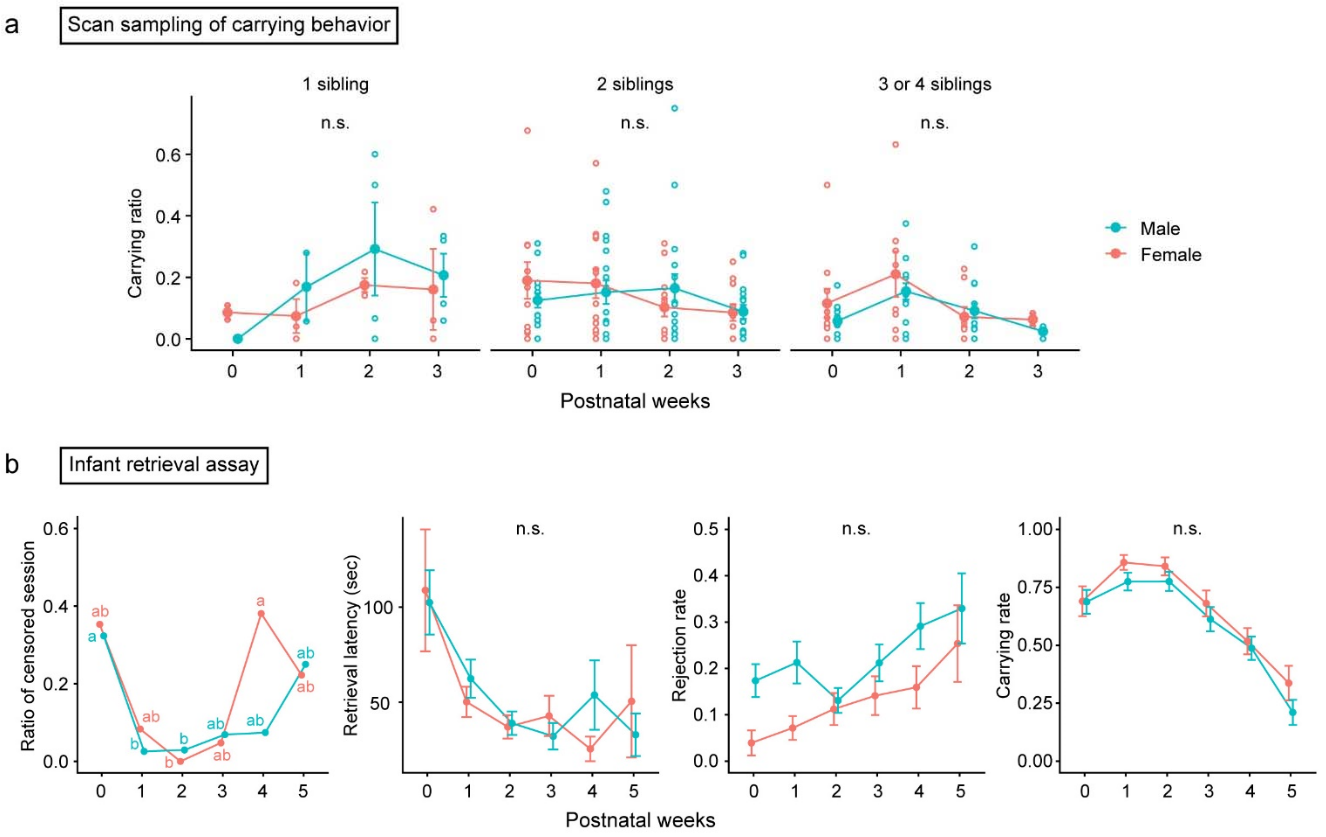

Supplementary Fig. 1 Sex difference in siblings' caregiving behavior.

(a) Siblings' carrying ratio (mean ± SEM) in the scan sampling (male: n = 20, female: n = 14, corresponds to orange lines in Fig. 1i-k). No significant effect in sex or an interaction of sex and PNW was observed (LMM).

(b) Siblings' performance on the infant retrieval assay (male: n = 19, female: n = 13, corresponds to orange lines in Fig. 3d-g). Male and female siblings showed similar changes in ratio of censored session, while females did not reach significance due to a smaller number of samples. Different alphabets indicate statistically-significant differences (Fisher's exact test, $p < 0.05$). All of retrieval latency, rejection rate, and carrying rate (mean ± SEM) did not differ between male and female siblings (LMM).

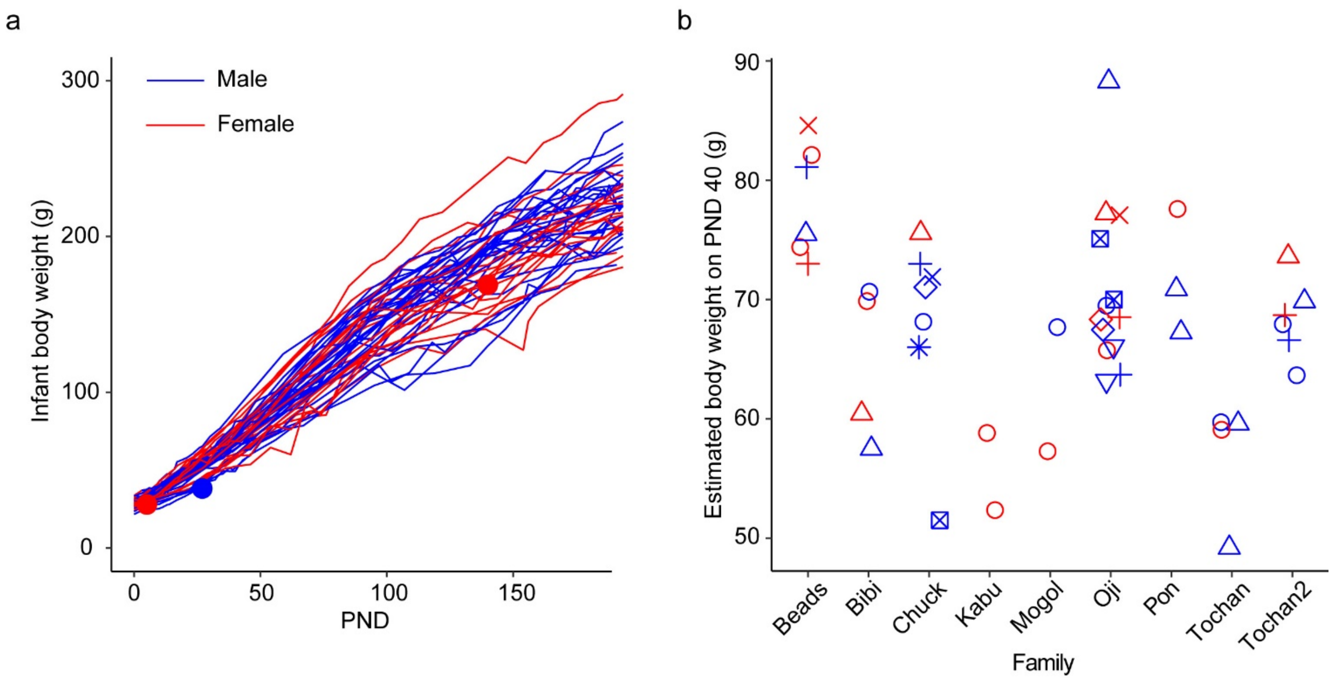

Supplementary Fig. 2 Infant growth.

(a) A growth curve for 49 infants until PND 180. Individuals died within this period were shown in large dots. Blue and red lines indicated male and female infants.

(b) An estimated body weight of infants on PND 40 in each family. These body weights were calculated as linear interpolation of two nearest points. Same-litter siblings were indicated as same markers. Blue and red indicated male and female infants.

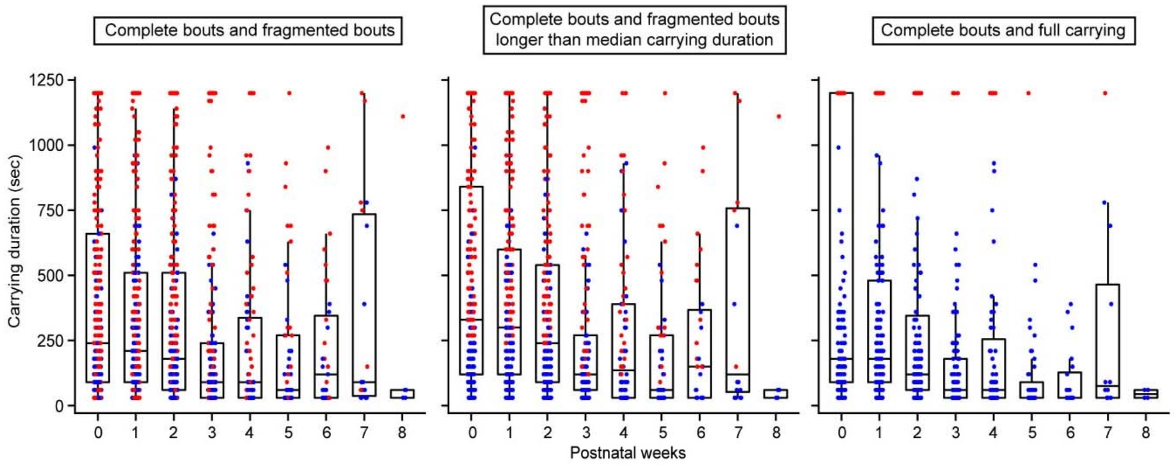

Postnatal weeks	# of sessions	# of bouts	Mean duration	# of bouts	Mean duration	# of bouts	Mean duration
0	116	309	424	256	487	170	435
1	98	312	363	253	423	169	354
2	84	292	343	249	389	175	324
3	49	191	221	162	252	126	189
4	46	104	250	86	289	63	234
5	39	63	183	61	188	47	115
6	42	35	225	32	241	20	100
7	38	18	357	16	396	12	290
8	14	5	258	5	258	4	45

Supplementary Fig. 3 Carrying duration in family observation.

An approximate carrying duration in all carrying bouts (1329) were shown (left). Horizontal lines indicated median, top and bottom ends of boxes indicated 25th and 75th percentiles, and top and bottom ends of vertical bars indicated maximum and minimum data points within 1.5 × inter-quartile range. These observations include carrying bouts without onset and/or without offset, so that mean duration was underestimated than actual carrying behavior. Mean duration with no onset/offset bouts longer than median duration (middle) and mean duration without no onset/offset bouts except for full carrying (right) were also shown. Mean duration with no onset/offset bouts longer than median duration were used for analysis in Fig. 2b, 2d, and 4e. (Blue dots; complete bouts, red dots; no onset/offset bouts)

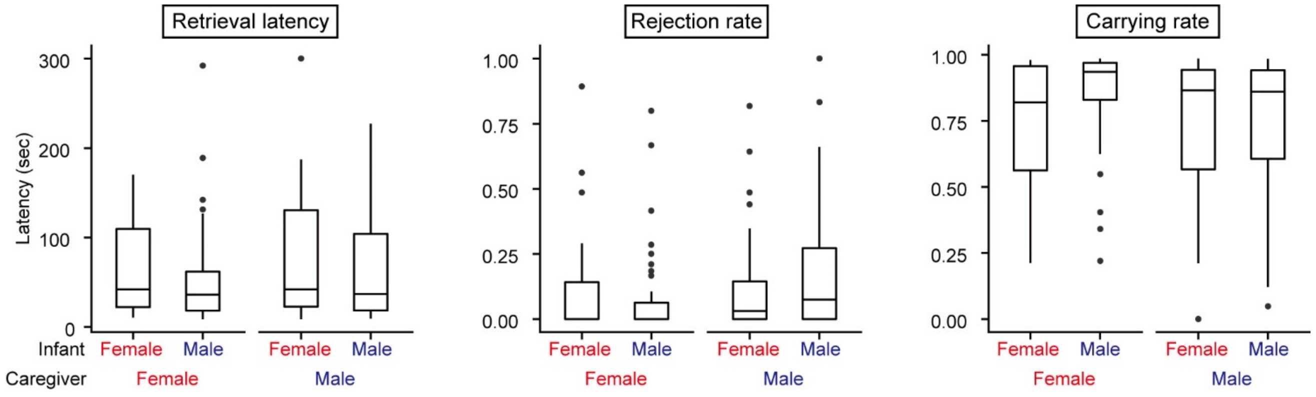

Supplementary Fig. 4 Effects of caregiver's and infant's sex on the infant retrieval assay.

Results of the infant retrieval assay were plotted over caregiver's and infant's sex. Horizontal lines indicated median, top and bottom ends of boxes indicated 25th and 75th percentiles, and top and bottom ends of vertical bars indicated maximum and minimum data points within 1.5 × inter-quartile range. No significant effect of infant's sex, caregiver's sex, and interaction between them was found for each index by LMM.

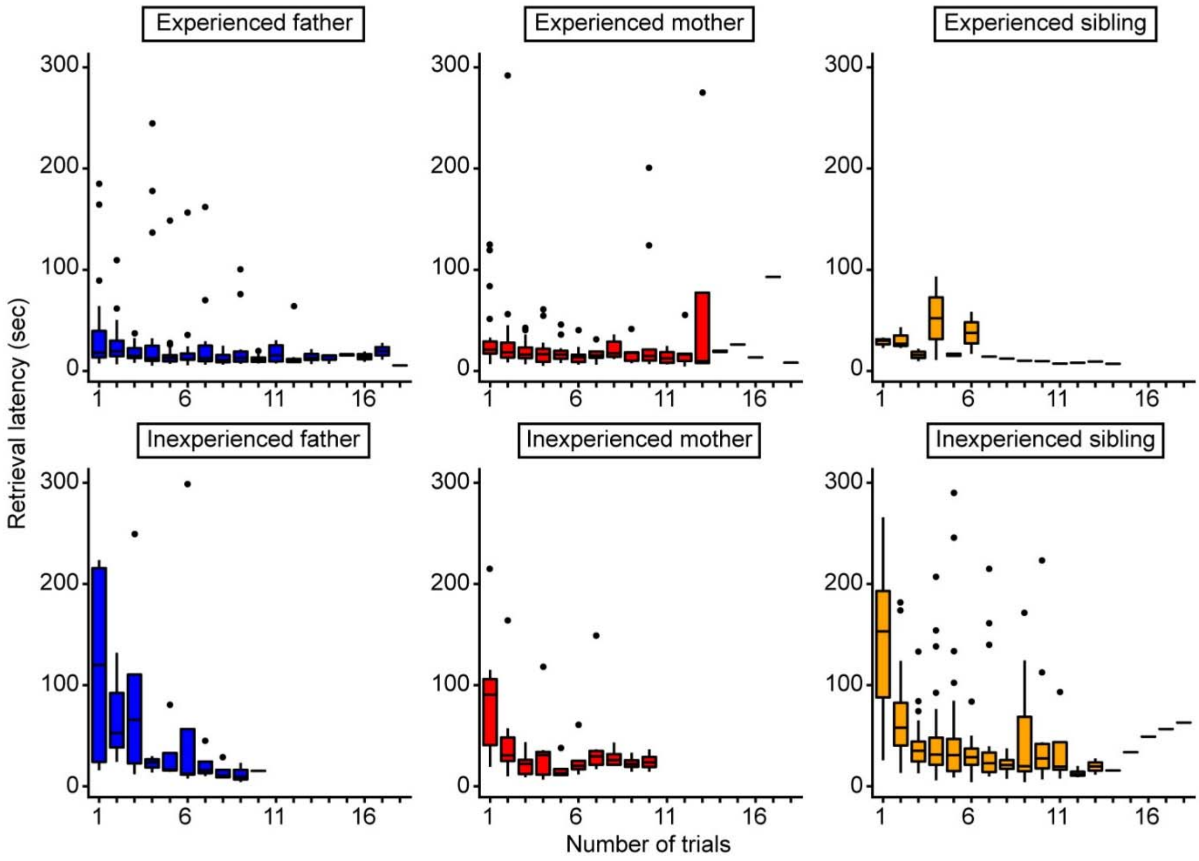

Supplementary Fig. 5 Effects of the experience of the infant retrieval assay on retrieval latency. A retrieval latency in the infant retrieval assay for fathers, mothers, and siblings was shown. Horizontal lines indicated median, top and bottom ends of boxes indicated 25th and 75th percentiles, and top and bottom ends of vertical bars indicated maximum and minimum data points within 1.5 × inter-quartile range. Experienced animals who had experience of the infant retrieval assay in previous birth showed constantly short latency from initial trials. In contrast, inexperienced animals who received the infant retrieval assay for the first time in this birth showed longer retrieval latency in initial trials and gradual improvement, suggesting leaning of skills to retrieve an infant from the basket.

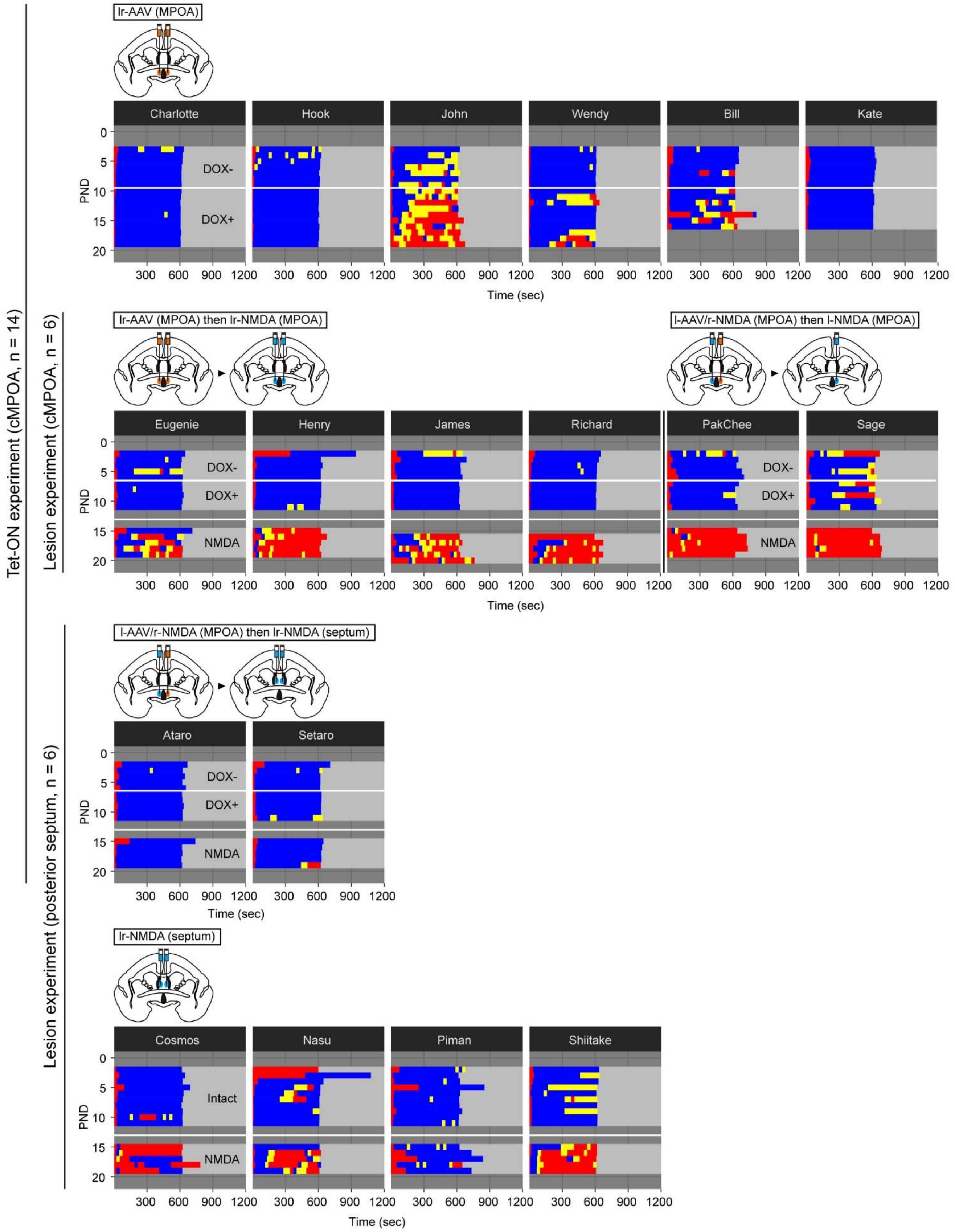

Supplementary Fig. 6 List of surgical interventions with result of the infant retrieval assay.

In total, 18 older siblings received injection of AAV and/or NMDA into the MPOA or the septum. First, as the Tet-ON experiment ($n = 14$), 10 subjects received bilateral AAV injections, and 4 subjects received left-AAV and right-NMDA injections expected to facilitate TeNT effect in virus injected side. Then as the lesion experiment, 4 bilateral AAV and 2 l-AAV/r-NMDA injected subjects received NMDA injection

into the MPOA (cMPOA lesion group, n = 6). Also, 2 l-AAV/r-NMDA injected and 4 intact subjects received NMDA injection into the septum (posterior septum lesion group, n = 6). All results of the infant retrieval assay in these subjects were shown as raster plots. blue, the subject carried the infant at the beginning of the bin; yellow, the bins of which beginning the caregiver carried the infant but rejected it within the bin; and red, the subject did not carry the infant at the beginning of the bin, or before the initial retrieval. DOX- and DOX+ phase indicated Tet-ON experiment. NMDA phase indicated lesion experiment.

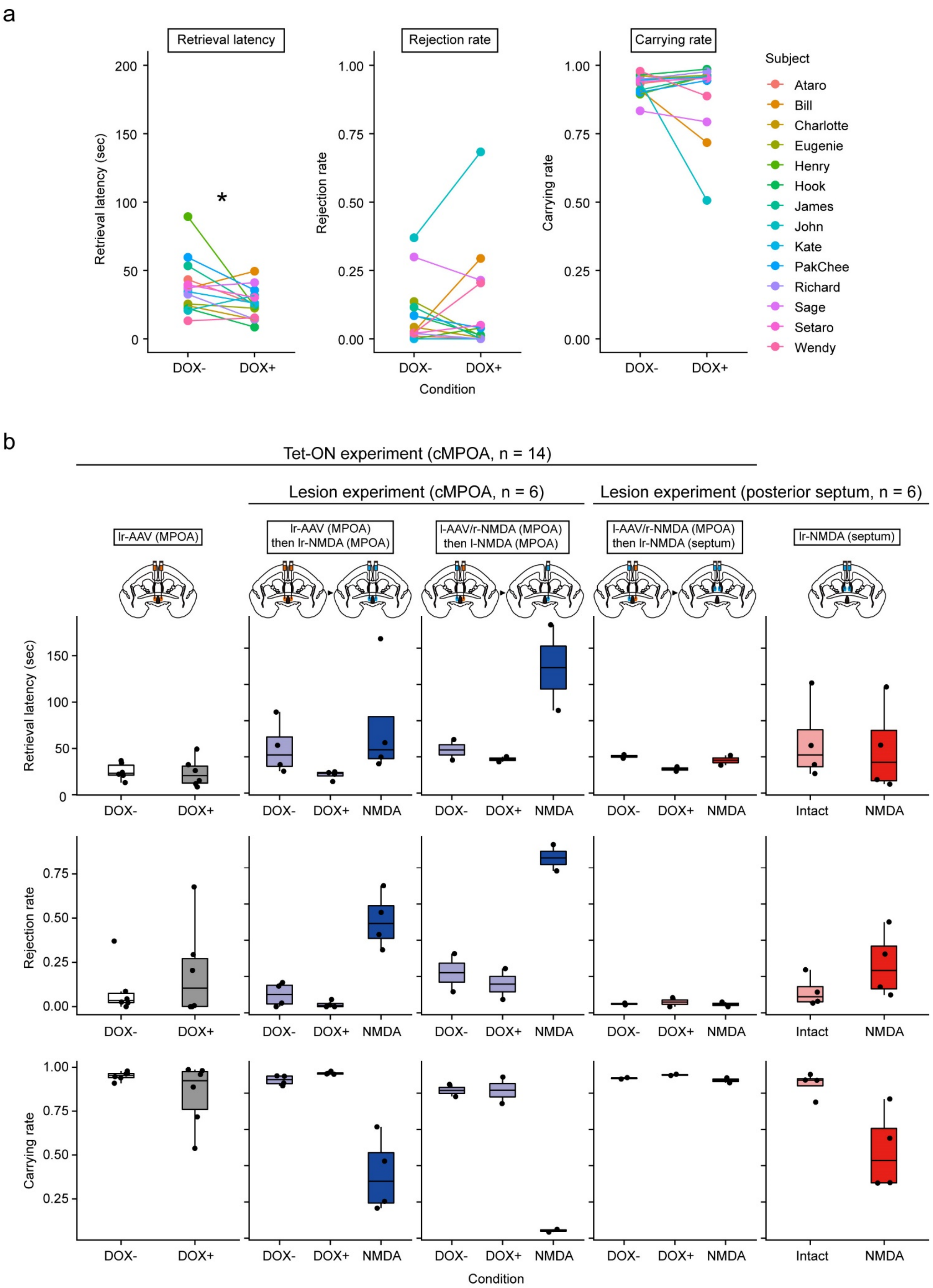

Supplementary Fig. 7 Infant retrieval assay before and after tetanus toxin expression in MPOA by Tet-ON system.

AAV-born TeNT and rtTA were introduced into the subjects' MPOA (bilateral AAV: *n* = 10, unilateral AAV and NMDA: *n* = 4).

(a) Before and after daily doxycycline administration, the infant retrieval assay was conducted. Rejection rate and carrying rate did not change. Retrieval latency is significantly reduced (improved), instead of increased. Reduction of retrieval latency was also seen in intact siblings (Fig. 3e) and inexperienced fathers and mothers (Supplementary Fig. 5), suggesting this improvement was due to skill learning for this task rather than effect of induction of TeNT by DOX administration. (Welch's paired t-test, * $p < 0.05$)

(b) Results of the infant retrieval assay divided by all surgical interventions. Horizontal lines indicated median, top and bottom ends of boxes indicated 25th and 75th percentiles, and top and bottom ends of vertical bars indicated maximum and minimum data points within 1.5 × inter-quartile range. There was no obvious difference among surgical groups during DOX- and DOX+ periods. For lesion experiment shown in Fig. 6, surgical groups in each lesion group were merged, then DOX- and DOX+ periods were pooled as pre-lesion period.

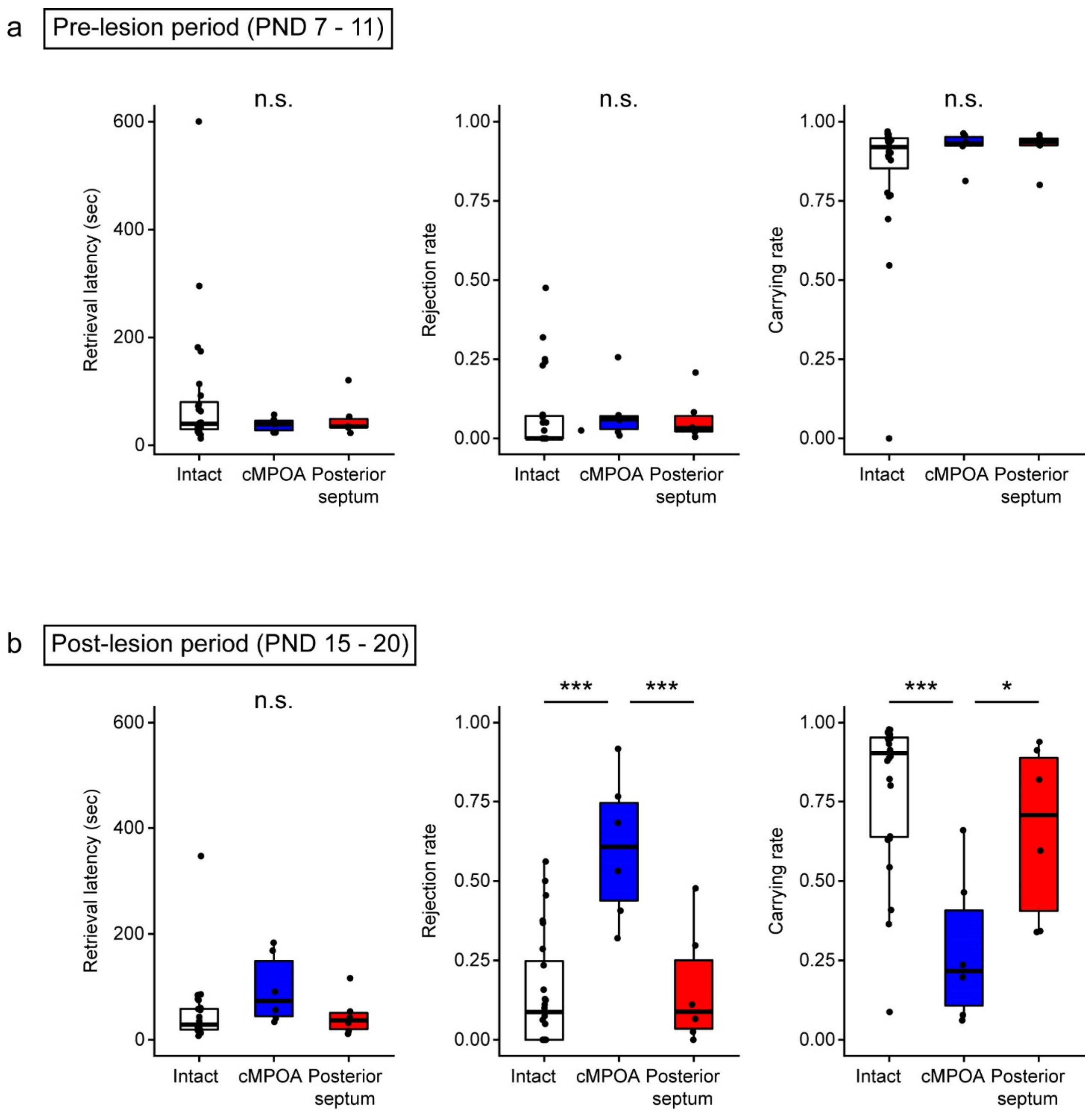

Supplementary Fig. 8 Comparisons among intact and lesioned subjects

Performance of the infant retrieval assay in cMPOA-lesion group (n = 6, 3 males and 3 females) and posterior septum-lesion group (n = 6, 5 males and 1 female) were compared with that of intact siblings (n = 24 consisted of 14 males and 10 females, a subset of dataset in Fig. 3; Siblings that had at least one data-point within both pre- and post-lesion periods were selected.) Twenty-three out of 24 intact siblings had not had an experience with the infant retrieval assay in the previous birth. For comparison, data until PND 7 in the intact group were excluded from this analysis to remove the initial learning effect in inexperienced subjects (see Supplementary Fig. 5). Horizontal lines indicated median, top and bottom ends of boxes indicated 25th and 75th percentiles, and top and bottom ends of vertical bars indicated maximum and minimum data points within 1.5 × inter-quartile range.

(a) Pre-lesion period. All of retrieval latency, rejection rate, and carrying rate did not show significant effect of the group.

(b) Post-lesion period. Rejection rate was significantly higher in the cMPOA group than the other groups. Carrying rate was significantly lower in the cMPOA group than the other groups. Retrieval latency did not differ among the group (general linear model, *** $p < 0.001$, * $p < 0.05$).

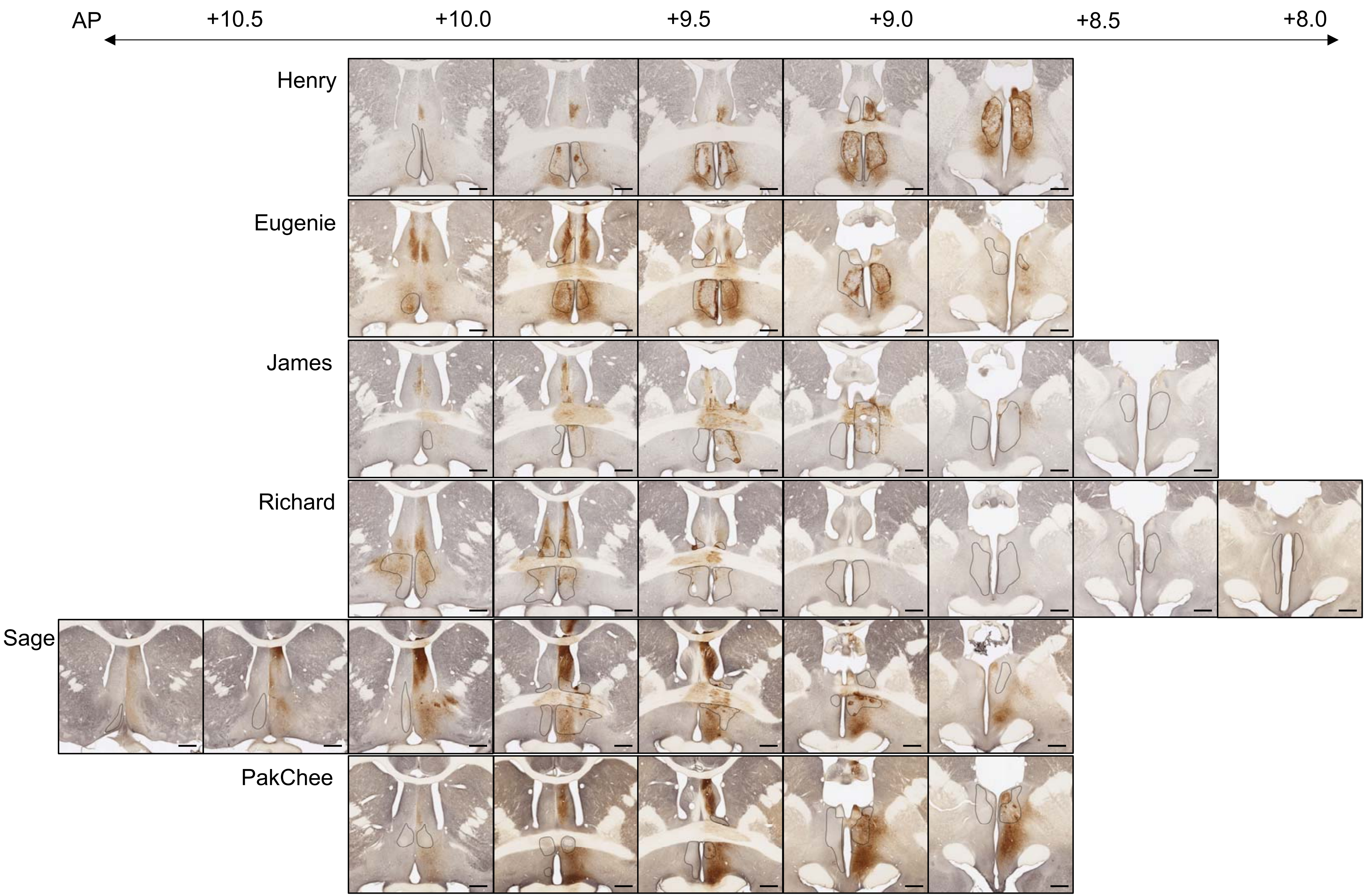
AP
+10.5
+10.0
+9.5
+9.0
+8.5
+8.0
Henry
Eugenie
James
Richard
Sage
PakChee

Supplementary Fig. 9 Histological evaluation of NMDA lesions for cMPOA-lesion group.
An immunohistochemical staining of coronal brain slices for the cMPOA-lesion group. All animals were also injected AAV to induce tetanus toxin (see methods for details, Black: NeuN, brown: TeNT.EGFP). Lesion areas were shown with bounding outline. Sections were arranged from anterior (left) to posterior (right). A top scale indicated approximate position from interaural (mm). Scale bars: 1 mm.

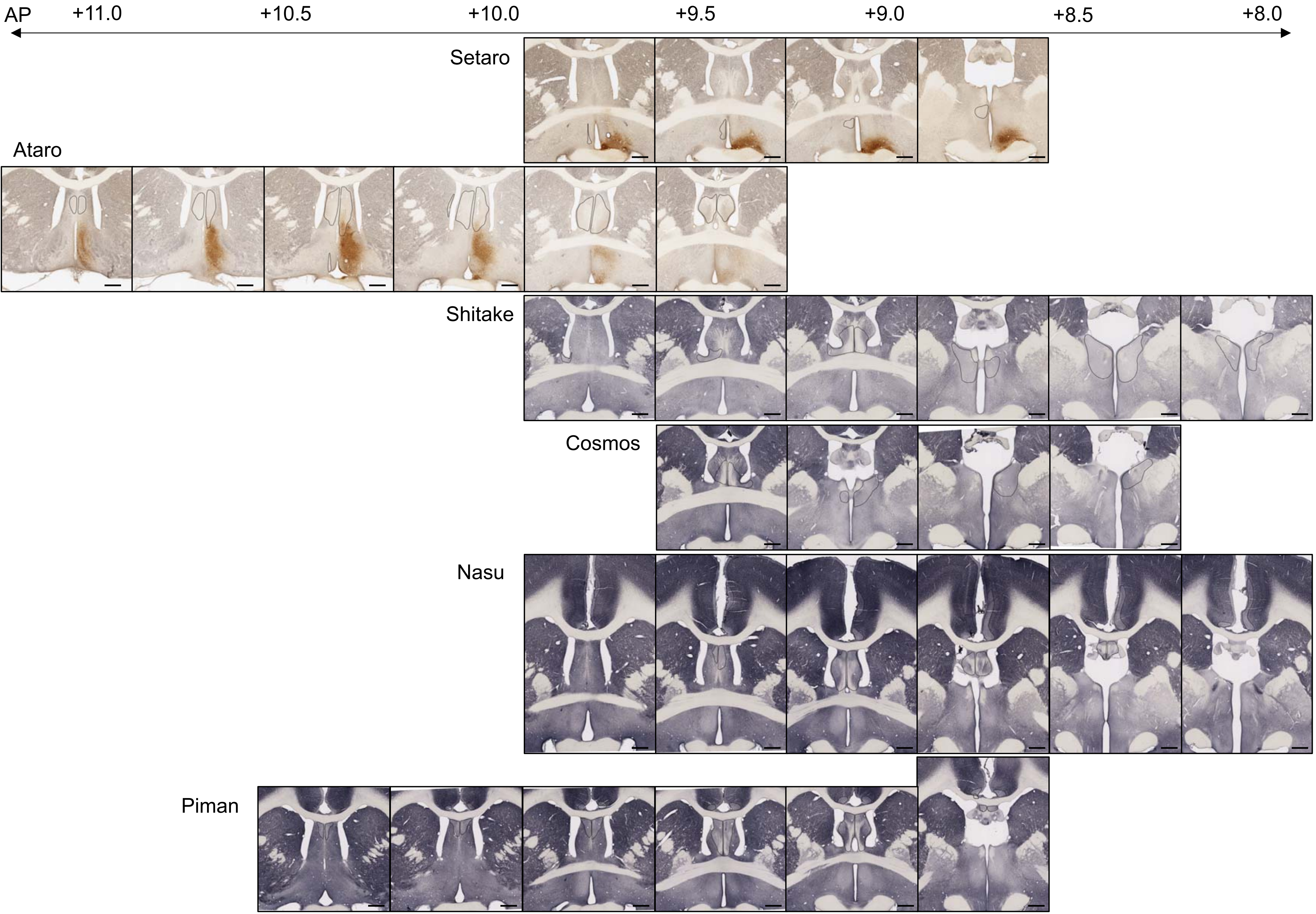

AP
+11.0
+10.5
+10.0
+9.5
+9.0
+8.5
+8.0
Setaro
Ataro
Shitake
Cosmos
Nasu
Piman

Supplementary Fig. 10 Histological evaluation of NMDA lesions for posterior septum-lesion group. An immunohistochemical staining of coronal brain slices for the posterior septum-lesion group. First two animals were also injected AAV to induce tetanus toxin (see methods for details, Black: NeuN, brown: TeNT.EGFP). Lesion areas were shown with bounding outline. Sections were arranged from anterior (left) to posterior (right). A top scale indicated approximate position from interaural (mm). Scale bars: 1 mm.

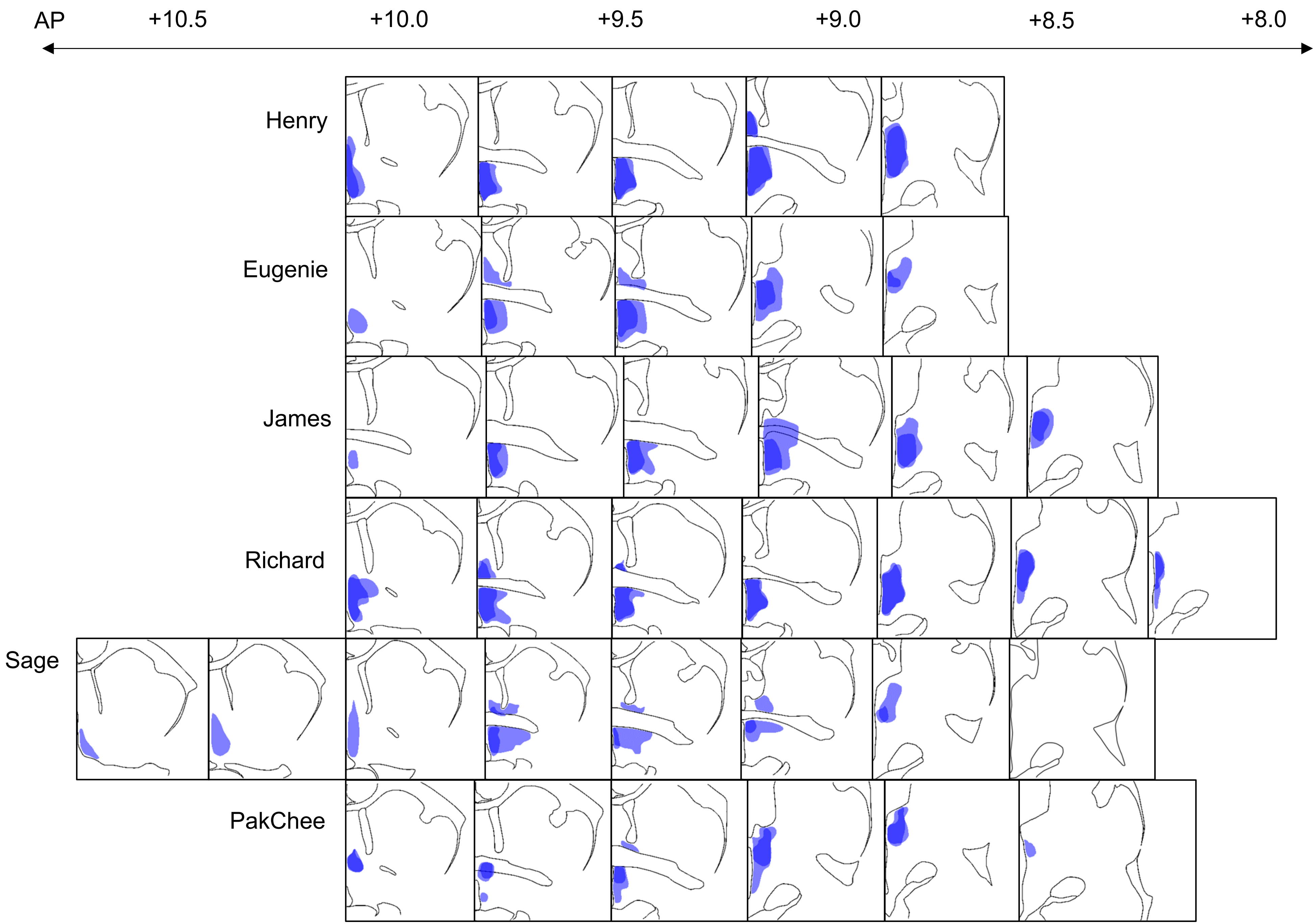

AP
+10.5
+10.0
+9.5
+9.0
+8.5
+8.0
Henry
Eugenie
James
Richard
Sage
PakChee

Supplementary Fig. 11 Bilateral lesion area for cMPOA-lesion group.

Coronal line drawings showed overlapped lesion area between left and right side. Light blue indicates lesion area at least in one side, while dark blue indicates lesion area in both sides. Sections were arranged from anterior (left) to posterior (right). A top scale indicated approximate position from interaural (mm).

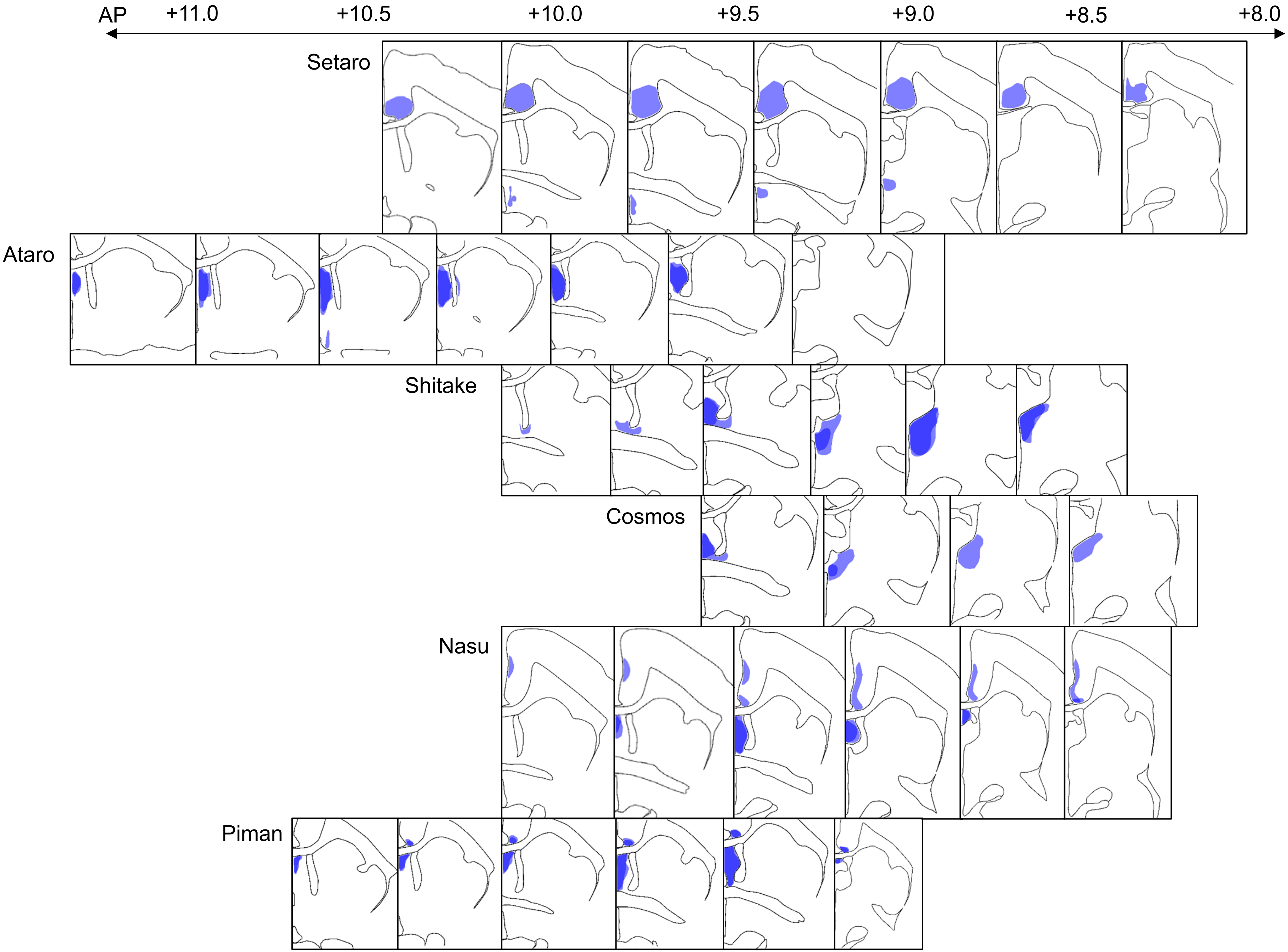
AP
+11.0
+10.5
+10.0
+9.5
+9.0
+8.5
+8.0
Setaro
Ataro
Shitake
Cosmos
Nasu
Piman

Supplementary Fig. 12 Bilateral lesion area for posterior septum-lesion group.
Coronal line drawings showed overlapped lesion area between left and right side. Light blue indicates lesion area at least in one side, while dark blue indicates lesion area in both sides. Sections were arranged from anterior (left) to posterior (right). A top scale indicated approximate position from interaural (mm).

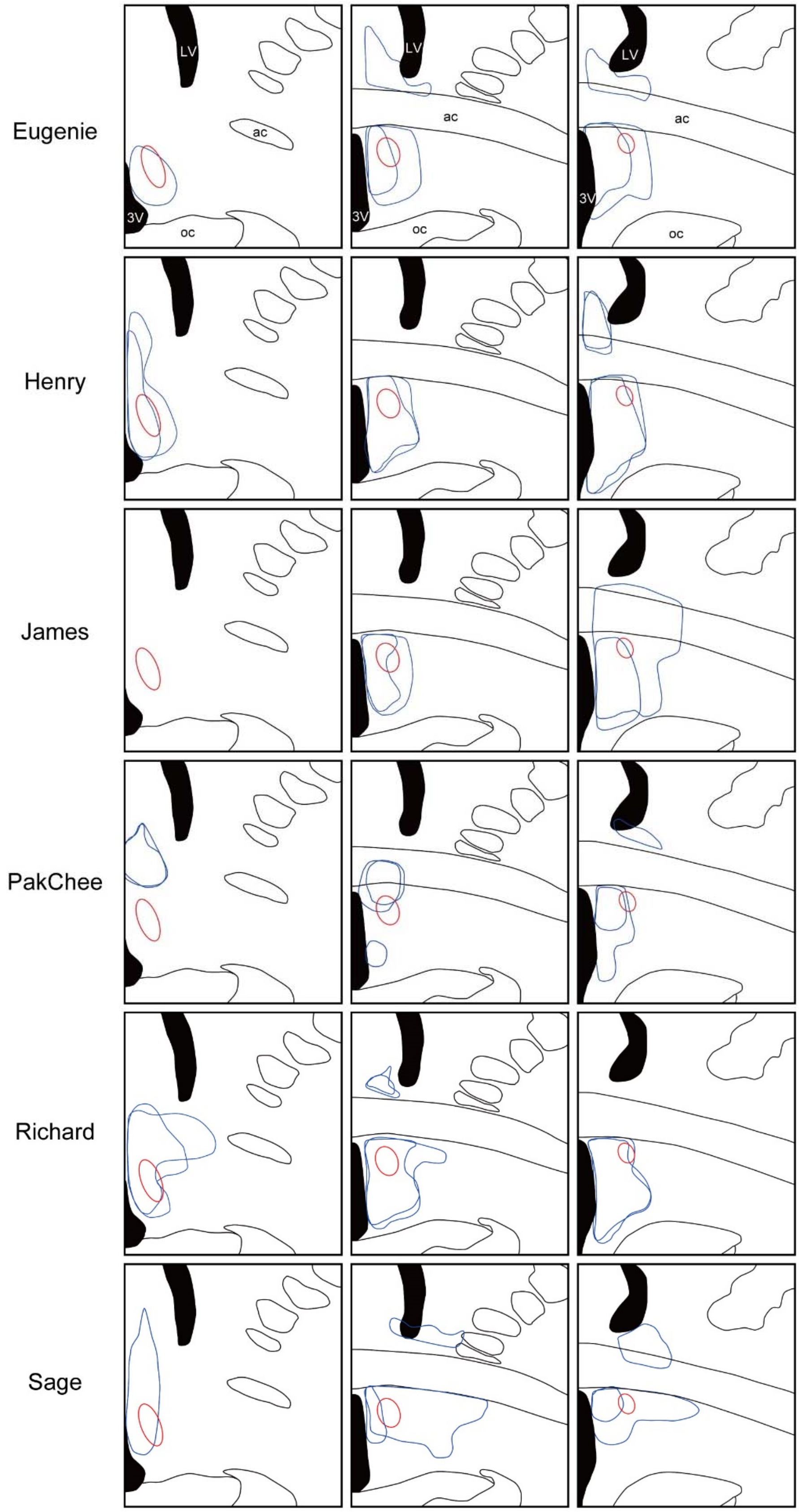

Supplementary Fig. 13 cMPOA lesion area

A contour of lesion area on each side and cMPOA was overlapped for three levels of brain sections for each subject. % lesion of cMPOA was calculated for each side for each section (Supplementary Table 1). Blue lines: lesion area for left and right brain. Red lines: conservative contour of cMPOA.

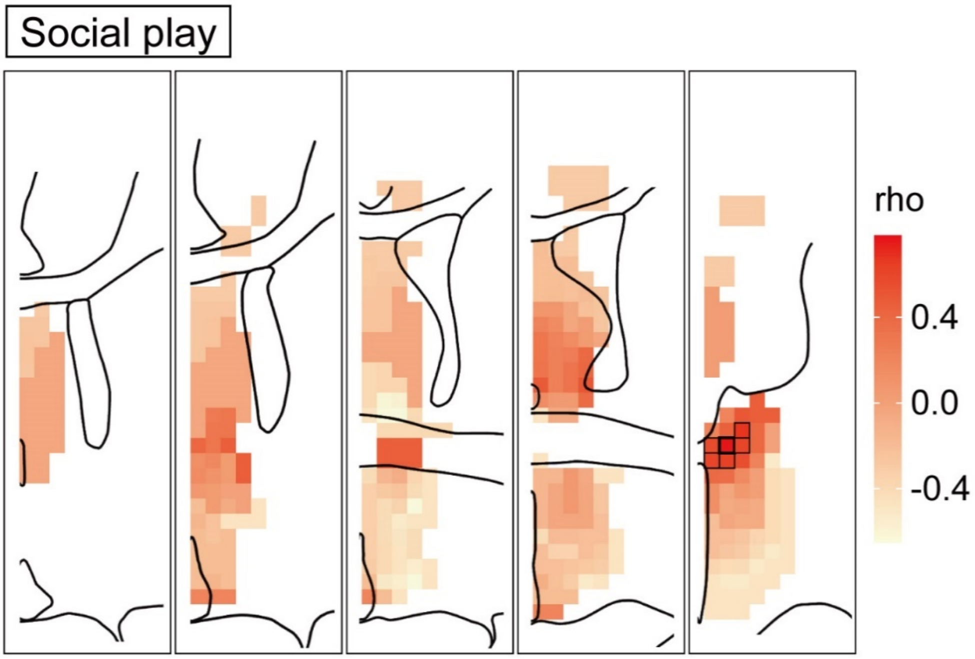

Supplementary Fig. 14 Grid-based correlations for social play

Grid-based correlations between the degree of cell loss relative to the same grid in the intact animal and the occurrence rate of the social play after surgery. Each grid is 250 μm × 250 μm. The darker grid denotes higher correlation between the behavior and damage. Grids with black frame: $p < 0.05$; grids with bold black frame: $p < 0.01$. (Spearman's rank correlation coefficients, $n = 12$ including both the cMPOA and posterior septum groups.)
