# Supplementary material for: A calcitonin receptor-expressing subregion of the medial preoptic area is involved in alloparental tolerance in common marmosets
Source: Commun Biol. 2022 Nov 21;5:1243. doi: 10.1038/s42003-022-04166-2 (PMC9678893; doi:10.1038/s42003-022-04166-2)
Supplement: Supplementary file 2 — Description of Additional Supplementary Files [file 42003_2022_4166_MOESM2_ESM.pdf]

Description of Additional Supplementary Files

File name: Supplementary Data 1
Description: List of experiments. Data were obtained from 10 families including 37 births. All of family members participated in observations and behavioral assays. Older siblings which were typically around 10 month-old served as subjects for virus/NMDA injection into cMPOA/posterior septum or analysis for c-Fos expression after an infant exposure. Two same-male, different female breeding pairs are counted separately. Chuck/Fastener's second row was not numbered because of the immediate death of the neonate. Colored cells in Days to next birth indicated pregnancy not at the immediately-following postpartum ovulation. Colored cells in Older/Younger siblings indicated subjects with surgical interventions; Orange, AAV injection into the cMPOA; Yellow, AAV and then NMDA injections into the cMPOA; Green, AAV injection into the cMPOA then NMDA injection into the posterior septum; Light green, NMDA injection into the posterior septum; Blue, cFos expression analysis with an infant exposure within a week of mother's delivery; Purple, cannula implantation for the other study. Note than Rin (light blue) received NMDA injection into the cMPOA but it was excluded from the analysis due to different experimental timeline as a pilot study. In Infant 1 to Infant 4, square and curly brackets indicated deaths found with injury or without injury, respectively (whether injury preceded death or not was unclear). Numbers indicate the PND of death. Angle brackets indicated assignment to other research immediately after birth. Tail indicated state of tale-eating. 0: intact tail, 1: less than half of tail eaten, 2: more than half of tail eaten. (M) and (F) indicated male and female, respectively. Gray cells in Scan sampling (carrying behavior) to RET assay (NMDA) were those not included in data analysis. Numbers indicated number of sessions included to the analysis. Parentheses in the Infant retrieval assay and the Food transfer assay indicated excluded sessions from analysis in Figure 3 and 4 because these sessions were conducted with subjects that received surgical interventions.

File name: Supplementary Data 2
Description: List of subjects and details of experimental interventions. All experimental interventions to marmosets were listed. PND indicates postnatal days of newborn infant(s) at the intervention. The minus values in dates indicate the days before the day of delivery of the mother.

File name: Supplementary Data 3
Description: The source data behind the graphs in the paper.

File name: Supplementary Movie 1
Description: An example video of the infant retrieval assay. A subject mother (Hime) started from a left cage and entered into a right cage through a tunnel. A stimulus infant (Diana, PND 10) in a basket was retrieved and carried on her back. A bottom part indicated a spectrogram of infant vocalizations. The infant initially emitted distress vocalizations but stopped in response to the retrieval.

File name: Supplementary Movie 2
Description: An example video of the rejection and reretrieval in the infant retrieval assay. A subject father (Cubby) started to reject an infant (Muroran, PND 6) on his back. The infant emitted vigorous vocalizations and finally got off from the caregiver. The infant kept vocalizing until she was carried again.

File name: Supplementary Movie 3
Description: An example video of the successful transfer in the food transfer assay. A subject mother

(Hime) took a piece of sweet potato. Then a juvenile (George, PND 96) got sweet potato from her hand and ate it. The subject did not show any refusal behavior.

File name: Supplementary Movie 4
Description: An example video of the refusal in the food transfer assay. A subject father (Chuck) did not transfer a piece of sweet potato to a juvenile (Wendy, PND 99) that showed an interest to the food item. The father showed refusal behavior such as away from the juvenile, emitting threat vocalization, and pushing.
