# Supplementary material for: A calcitonin receptor-expressing subregion of the medial preoptic area is involved in alloparental tolerance in common marmosets
Source: Commun Biol. 2022 Nov 21;5:1243. doi: 10.1038/s42003-022-04166-2 (PMC9678893; doi:10.1038/s42003-022-04166-2)
Supplement: Supplementary file 10 — Reporting Summary [file 42003_2022_4166_MOESM10_ESM.pdf]

nature portfolio

Corresponding author(s): Atsuko Saito and Kumi O. Kuroda

Last updated by author(s): Sep 16, 2022

Reporting Summary

Nature Portfolio wishes to improve the reproducibility of the work that we publish. This form provides structure for consistency and transparency in reporting. For further information on Nature Portfolio policies, see our Editorial Policies and the Editorial Policy Checklist.

Statistics

For all statistical analyses, confirm that the following items are present in the figure legend, table legend, main text, or Methods section.

n/a	Confirmed	
☐	☒	The exact sample size (*n*) for each experimental group/condition, given as a discrete number and unit of measurement
☐	☒	A statement on whether measurements were taken from distinct samples or whether the same sample was measured repeatedly
☐	☒	The statistical test(s) used AND whether they are one- or two-sided<br>*Only common tests should be described solely by name; describe more complex techniques in the Methods section.*
☐	☒	A description of all covariates tested
☐	☒	A description of any assumptions or corrections, such as tests of normality and adjustment for multiple comparisons
☐	☒	A full description of the statistical parameters including central tendency (e.g. means) or other basic estimates (e.g. regression coefficient) AND variation (e.g. standard deviation) or associated estimates of uncertainty (e.g. confidence intervals)
☐	☒	For null hypothesis testing, the test statistic (e.g. *F*, *t*, *r*) with confidence intervals, effect sizes, degrees of freedom and *P* value noted<br>*Give P values as exact values whenever suitable.*
☒	☐	For Bayesian analysis, information on the choice of priors and Markov chain Monte Carlo settings
☒	☐	For hierarchical and complex designs, identification of the appropriate level for tests and full reporting of outcomes
☐	☒	Estimates of effect sizes (e.g. Cohen's *d*, Pearson's *r*), indicating how they were calculated

Our web collection on statistics for biologists contains articles on many of the points above.

Software and code

Policy information about availability of computer code

Data collection	For behavioral analysis, video cameras (HDR-AS100V, Sony), a microphone (MKH416-P48U3, Sennheiser) and a linear PCM recorder (DR-60DMKII, TASCAM) were used for video and sound recording. For microscopy, image data was taken by NanoZoomer Digital Pathology (Hamamatsu Photonics) and an inverted microscope (KEYENCE BZ-X700, Osaka, Japan).
Data analysis	Statistics were performed using Excel 2019 and software R (R Development Core Team, 2021), and SPSS (IBM). Histochemical data was analyzed by ImageJ (version 1.52a, NIH).

For manuscripts utilizing custom algorithms or software that are central to the research but not yet described in published literature, software must be made available to editors and reviewers. We strongly encourage code deposition in a community repository (e.g. GitHub). See the Nature Portfolio guidelines for submitting code & software for further information.

Data

Policy information about availability of data

All manuscripts must include a data availability statement. This statement should provide the following information, where applicable:

- Accession codes, unique identifiers, or web links for publicly available datasets
- A description of any restrictions on data availability
- For clinical datasets or third party data, please ensure that the statement adheres to our policy

The authors declare that all data supporting the findings of this study are available within the paper and its supplementary information files.

Field-specific reporting

Please select the one below that is the best fit for your research. If you are not sure, read the appropriate sections before making your selection.

☒ Life sciences ☐ Behavioural & social sciences ☐ Ecological, evolutionary & environmental sciences

For a reference copy of the document with all sections, see nature.com/documents/nr-reporting-summary-flat.pdf

Life sciences study design

All studies must disclose on these points even when the disclosure is negative.

Sample size	Sample sizes were determined empirically, rather than statistically. We made an effort to reduce number of subjects as a primate study. Sample size for each experiment was described in the figure legends.
Data exclusions	All the data listed in Table S1 were included.
Replication	All the data were from at least two independent subsets of experiments, found to show a similar trend, and thus joined to the full sample. All attempts at replication were successful. The biological replicate number corresponds to the number of marmosets. Technical replicates were not performed in this study. Individual data is presented for transparency.
Randomization	Experiments and observations were conducted with several different marmoset families. For lesion experiment, subjects were randomly assigned to experimental groups.
Blinding	Most behavioral data were obtained on-site by the experimenter so that blinding was not applicable at that time. However, 20 % of the data were further analyzed by another experimenter blindly, and yielded high concordance rate. Histological analyses were not blinded, due to the high complexity of the tasks.

Reporting for specific materials, systems and methods

We require information from authors about some types of materials, experimental systems and methods used in many studies. Here, indicate whether each material, system or method listed is relevant to your study. If you are not sure if a list item applies to your research, read the appropriate section before selecting a response.

Materials & experimental systems

n/a	Involved in the study	
☐	☒	Antibodies
☒	☐	Eukaryotic cell lines
☒	☐	Palaeontology and archaeology
☐	☒	Animals and other organisms
☒	☐	Human research participants
☒	☐	Clinical data
☒	☐	Dual use research of concern

Methods

n/a	Involved in the study	
☒	☐	ChIP-seq
☒	☐	Flow cytometry
☒	☐	MRI-based neuroimaging

Antibodies

Antibodies used	anti-c-Fos (1:5,000, sc-52, Santa Cruz Biotechnology), anti-galanin antibody (1:5000, T-4334, Peninsula Laboratories International, Inc.), anti-NeuN antibody (1:8000, MAB377, Millipore), anti-Calcr antibody (1:400, AHP635, Bio-Rad), anti-Flag antibody (1:250, F1804, Sigma-Aldrich), anti-GFP antibody (1:10000, Code No. 598, MBL), OXT, AVP, biotin-conjugated horse anti-rabbit secondary antibody (1:2000, BA-1100, Vector Laboratories, Inc.), biotin-conjugated horse anti-goat secondary antibody (1:2000, BA-9500, Vector Laboratories), peroxidase-conjugated anti-digoxigenin (1:10000, Roche Diagnostics).
Validation	The antibodies used in this study are validated by the manufacturers as shown in the following websites.<br><br>anti-c-Fos https://www.scbt.com/p/c-fos-antibody-4?productCanUrl=c-fos-antibody-4&_requestid=12133601<br>anti-galanin http://www.bma.ch/en/products/t-4334<br>anti-NeuN https://www.merckmillipore.com/JP/ja/product/Anti-NeuN-Antibody-clone-A60,MM_NF-MAB377<br>anti-Calcr https://www.bio-rad-antibodies.com/polyclonal/rat-calcitonin-receptor-antibody-ahp635.html<br>anti-Flag https://www.sigmaaldrich.com/JP/ja/search/f1804?focus=products&page=1&perPage=30&sort=relevance&term=f1804&type=product_name<br>anti-GFP https://www.mblintl.com/products/598/<br>anti-NPI https://www.scbt.com/p/neurophysin-i-antibody-m-15<br>anti-AVP https://www.merckmillipore.com/JP/ja/product/Anti-Vasopressin-Antibody,MM_NF-AB1565<br>biotin-conjugated horse anti-rabbit secondary antibody https://vectorlabs.com/biotinylated-horse-anti-rabbit-igg-antibody.html

	biotin-conjugated horse anti-goat secondary antibody https://vectorlabs.com/biotinylated-horse-anti-goat-igg-antibody.html<br>peroxidase-conjugated anti-digoxigenin https://www.sigmaaldrich.com/JP/ja/product/roche/11633716001

Animals and other organisms

Policy information about studies involving animals; ARRIVE guidelines recommended for reporting animal research

Laboratory animals	Male and female common marmosets were used.
Wild animals	n.a.
Field-collected samples	n.a.
Ethics oversight	All experiments involving marmosets were performed in agreement with guidelines of the Animal Experiment Committee of RIKEN.

Note that full information on the approval of the study protocol must also be provided in the manuscript.
